# *Centrin*-deficient *Leishmania mexicana* confers protection against New World cutaneous leishmaniasis

**DOI:** 10.1038/s41541-022-00449-1

**Published:** 2022-03-02

**Authors:** Greta Volpedo, Thalia Pacheco-Fernandez, Erin A. Holcomb, Wen-Wei Zhang, Patrick Lypaczewski, Blake Cox, Rebecca Fultz, Chelsea Mishan, Chaitenya Verma, Ryan H. Huston, Abigail R. Wharton, Ranadhir Dey, Subir Karmakar, Steve Oghumu, Shinjiro Hamano, Sreenivas Gannavaram, Hira L. Nakhasi, Greg Matlashewski, Abhay R. Satoskar

**Affiliations:** 1 Department of Microbiology, The Ohio State University, Columbus, OH 43210 USA; 2 Department of Pathology, Wexner Medical Center, The Ohio State University, Columbus, OH 43210 USA; 3 Department of Microbiology and Immunology, McGill University, Montreal, QC Canada; 4 Division of Emerging and Transfusion Transmitted Diseases, CBER, FDA, Silver Spring, MD USA; 5 Department of Parasitology, Institute of Tropical Medicine (NEKKEN), The Joint Usage/Research Center on Tropical Disease, Nagasaki University, Nagasaki University Graduate School of Biomedical Sciences Doctoral Leadership Program, Nagasaki, Japan

**Keywords:** Live attenuated vaccines, Vaccines

## Abstract

Leishmaniasis is a neglected protozoan disease affecting over 12 million people globally with no approved vaccines for human use. New World cutaneous leishmaniasis (CL) caused by *L. mexicana* is characterized by the development of chronic non-healing skin lesions. Using the CRISPR/Cas9 technique, we have generated live attenuated *centrin* knockout *L. mexicana (LmexCen*^*−/−*^*)* parasites. Centrin is a cytoskeletal protein important for cellular division in eukaryotes and, in *Leishmania*, is required only for intracellular amastigote replication. We have investigated the safety and immunogenicity characteristics of *LmexCen*^*−/−*^ parasites by evaluating their survival and the cytokine production in bone-marrow-derived macrophages (BMDMs) and dendritic cells (BMDCs) in vitro. Our data shows that *LmexCen*^*−/−*^ amastigotes present a growth defect, which results in significantly lower parasitic burdens and increased protective cytokine production in infected BMDMs and BMDCs, compared to the wild type (WT) parasites. We have also determined the safety and efficacy of *LmexCen*^*−/−*^ in vivo using experimental murine models of *L. mexicana*. We demonstrate that *LmexCen*^*−/−*^ parasites are safe and do not cause lesions in susceptible mouse models. Immunization with *LmexCen*^*−/−*^ is also efficacious against challenge with WT *L. mexicana* parasites in genetically different BALB/c and C57BL/6 mouse models. Vaccinated mice did not develop cutaneous lesions, displayed protective immunity, and showed significantly lower parasitic burdens at the infection site and draining lymph nodes compared to the control group. Overall, we demonstrate that *LmexCen*^*−/−*^ parasites are safe and efficacious against New World cutaneous leishmaniasis in pre-clinical models.

## Introduction

*Leishmania (L.) mexicana* is the most prevalent causative agent of cutaneous leishmaniasis (CL) in North and Central America^1,2^. *L. mexicana* causes extensive skin lesions that, in some cases, further develop into diffuse sores on different parts of the body and can spread to the mucosa^1,2^. CL lesions can ulcerate and give rise to disfiguring scars, leading to long-lasting disabilities, social stigma, poverty, and psycho-sociological trauma^3–6^. These issues not only impact an individual’s health and quality of life but also result in an overall economic strain, especially for low- and middle-income countries (LMIC)^3,7,8^. Despite the high morbidity of CL, there are no approved human vaccines and the current therapeutic agents often present significant toxicity as well as increased parasitic resistance^9–11^.

A protective immune response against CL is characterized by the activation of phagocytes, such as macrophages and dendritic cells (DCs), and by the subsequent induction of Th1-polarized responses^12^. Interleukin (IL)-12, secreted by phagocytes, promotes differentiation of Th0 into Th1 cells and subsequent release of interferon-γ (IFN-γ), crucial for combating leishmaniasis^12^. While a Th1 immune response is mostly protective, a Th2 response, characterized by IL-10 and IL-4, confers susceptibility to CL^12–15^. An effective vaccine must induce a robust immune response, followed by the generation of T memory cell populations, some of which require antigen persistence to be maintained^16,17^. A live attenuated parasite will be able to persist longer than other types of vaccines, promoting a sustained exposure to *Leishmania* antigens, necessary to maintain effector and memory cell pools and mediate long-lasting protection^18^.

A variety of tools including homologous recombination, plasmid shuffle, and Cre recombinase, have been used in the past to genetically modify *Leishmania* parasites^19^. However, these techniques are time-consuming, require the use of an antibiotic resistance marker, and have limited efficiency and versatility^20^. Recently, different groups have been working on developing an *L. mexicana*-specific CRISPR/Cas9 toolkit to delete genes encoding different virulence factors^21–24^. Our group has previously generated live attenuated *L. major* mutants via CRISPR/Cas9-mediated knock out of the *centrin* 1 (*Cen*) gene^25^. We chose this advanced technique as it allows for targeted genetic manipulation without leaving behind a functional antibiotic-resistant gene that may affect the clinical deployment of such vaccines. *Cen* codes for a cytoskeletal protein important for eukaryotic cellular division, but is only necessary for amastigote replication in *Leishmania*^26^. Previous studies from our group indicate that CRISPR/Cas-generated *Cen* knockout (*Cen*^*−/−*^) *L. major* parasites elicit a protective immune response, reduce the parasitic burdens, and prevent symptoms in *L. major* and *L. donovani* infection models, showing potential as prophylactic vaccines^25,27^. Nevertheless, *L. major Cen*^*−/−*^ is derived from *L. major* Friedlin, a strain that is endemic to the Old World and presents different clinical characteristics and pathologies when compared to the New World strains, such as *L. mexicana*^28–30^. For example, *L. major* causes self-resolving localized skin lesions in humans, while *L. mexicana* can further develop into long-lasting diffuse lesions in distal parts of the body with an absent delayed-type hypersensitivity (DTH) response^28,29^. Susceptibility to *L. mexicana* is mediated by the induction of a Th2-dominant immune response in most mouse genetic backgrounds^31^. Additionally, in C57BL/6 murine models, *L. mexicana* infection disrupts IL-12 production in DCs^32^ and results in susceptibility mediated by impaired lymph node expansion and reduced Th1 differentiation^33^. On the other hand, inoculation with *L. major* leads to a sustained IL-12-driven Th1 protective response, and ultimately to the resolution of the infection in C57BL/6 mice^33^. Unlike for *L. major* infections, where protection against homologous species of *Leishmania* is observed in humans following cure, a phenomenon often referred to as leishmanization, there are currently no studies showing that leishmanization also confers protection against New World species such as *L. mexicana*. These observations highlight the importance of developing a vaccine strain specific for American cutaneous leishmaniasis.

In this study we generated *L. mexicana Cen*^*−/−*^ (*LmexCen*^*−/−*^) parasites, validated their safety and efficacy as a prophylactic vaccine against the endemic subspecies of *L. mexicana* in Central and South America, and determined the immunological mechanisms underlying long-lasting protection. *Cen*^*−/−*^ *Leishmania* parasites could become an efficacious leishmaniasis vaccine for human use, filling an important critical need worldwide.

## Results

### Generation of *centrin* deficient *L. mexicana* parasites with no remaining antibiotic selection marker activity

**Fig. 1:**
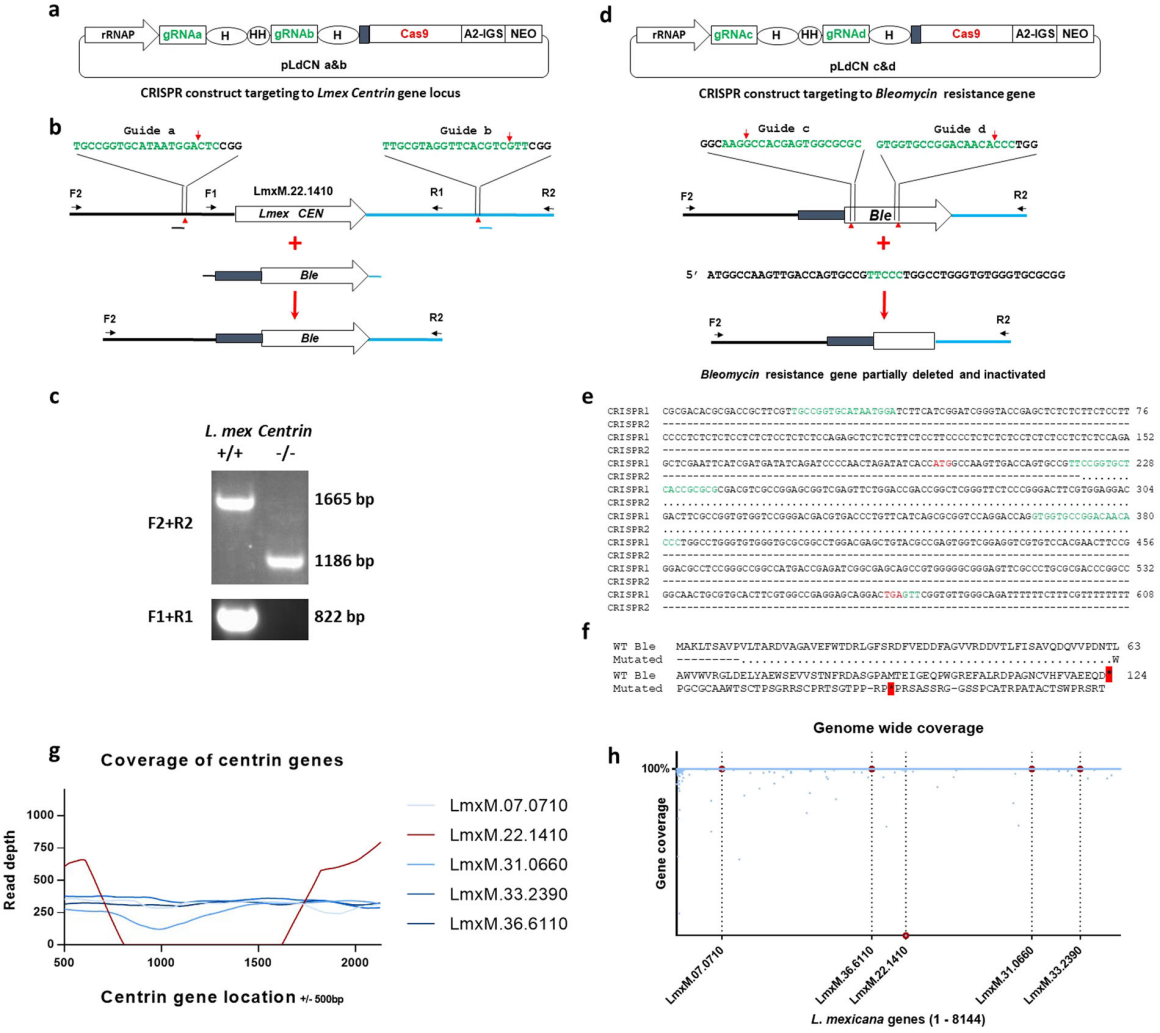
Generation of *LmexCen*^*−/−*^ null mutant with no antibiotic selection marker activity. **a** Scheme of the *Leishmania* CRISPR vector pLdCN a&b expressing the Cas9 nuclease and two gRNAs targeting the 5′ and 3′ flanking sequences of *L. mexicana centrin* gene. **b** The CRISPR strategy to delete *L. mexicana centrin* gene. The small filled black rectangle in front of Bleomycin resistance gene (*Ble*) represents a 92 bp pyrimidine track which is required for trans-splicing processing of *Ble* transcripts. **c** PCR analysis with primer pairs F1R1 and F2R2 verified that both alleles of *L. mexicana centrin* gene were deleted as planned. **d** The CRISPR strategy to partially delete and inactivate the *Ble* gene targeted in the *centrin* gene locus. **e** DNA sequencing revealed that 160 bp of *Ble* gene sequence has been deleted after CRISPR double gRNA targeting and oligo donor transfection. CRISPR1 first round of CRISPR targeting, CRISPR2 second round of targeting. The sequences in green are the partial remaining or the complete gRNA targeting sites. The start codon ATG and stop codon TGA for *Ble* gene are highlighted in Red. **f** Amino acids alignment shows the remaining *Ble* sequence in the *centrin* gene locus is only able to encode 9 amino acids identical to the wild-type *Ble* gene product; the 160 bp DNA sequence deletion also caused a frame shift resulting in non-mature stop codon. Dashed lines represent the remaining sequence to the WT *Ble* gene; Dot lines represent the deleted sequences. **g** Sequencing coverage across each of the five *centrin* gene family members and partial flanking UTRs in the *L. mexicana* gene edited strain. The CRISPR targeted LmxM.22.1410 gene coverage is shown in red, non-targeted *centrin* genes are shown in shades of blue. **h** Percent coverage (*y*-axis) of all genes across the *L. mexicana* genome (*x*-axis) is shown in blue. The location of the *centrin* gene family members is highlighted by a dotted line and the genes are colored in red.

Wildtype (WT) *L. mexicana* promastigotes were transfected with the pLdCN a&b CRISPR vector (Fig. 1a) containing a neomycin (G418)-resistant gene, and expressing the Cas9 nuclease and the two guide RNAs (gRNAs) a and b, targeting the 5′ and 3′ flanking sequences of the *centrin* gene (LmxM.22.1410) (Fig. 1b). Once the pLdCN a&b CRISPR vector-transfected culture was established following G418 selection, the promastigotes were further transfected with a 584 bp Bleomycin (Phleomycin) resistance gene (*Ble*) donor, followed by Phleomycin selection and cloning. PCR analysis on these Phleomycin resistance clones confirmed that both alleles of *L. mexicana centrin* gene were deleted as expected (Fig. 1c). Since centrin-deficient *Leishmania* cells could potentially be used as a live attenuated *Leishmania* vaccine for humans^25,27^, the *Ble* gene targeting the *centrin* locus in this *L. mexicana centrin* null mutant was subsequently inactivated with CRISPR (Fig. 1d). The *centrin* null mutant *Leishmania* cells were cultured in G418 free medium for several weeks to remove the pLdCN a&b plasmid. The cells were then re-transfected with the second CRISPR vector pLdCN c&d, which expresses gRNA c and d targeting the coding sequence of the *Ble* gene. To improve gene editing specificity and efficiency, G418 resistant *L. mexicana* parasites were subsequently transfected with a 49 nucleotide oligo donor followed by cloning these cells in duplicate 96 well plates with or without Phleomycin in the medium. Sequencing analysis of the clones, which were unable to grow in Phleomycin-containing plates confirmed that part (160 bp) of the *Ble* gene had been deleted as planned (Fig. 1d–e). Because the partial deletion also resulted in a frameshift and a new stop codon, the remaining *Ble* sequence in the *centrin* locus is only able to encode a 40 amino acid peptide which, due to the frameshift, retains only 9 amino acids of the 124 amino acids *Ble* gene product (Fig. 1f). Likewise, the pLdCN c&d CRISPR vector was subsequently removed from these *centrin* null mutants by culturing in G418 free medium.

To ensure the specificity of *centrin* deletion by CRISPR/Cas9, we performed complete genome sequencing of *LmexCen*^*−/−*^. Alignment of *LmexCen*^*−/−*^ sequence reads against the reference genome of *L. mexicana* (MHOM/GT/2001/U1103) revealed the absence of the *centrin* gene corresponding to LmxM.22.1410 (Fig. 1g, shown in red). Sequencing coverage across each of the five *centrin* gene family members showed that the non-targeted *centrin* genes remained unaffected (Fig. 1g, shown in blue). Genome-wide sequencing showed the coverage corresponding to the coding genes represented in the reference genome. This analysis also showed coverage of all *centrin* genes, except for the target *centrin* gene LmxM.22.1410 (Fig. 1h, red circles). Taken together, this data shows that CRISPR based targeting results in an *L. mexicana centrin* null mutant with no untargeted mutations and no remaining antibiotic selection marker activity.

### *LmexCen*^*−/−*^ amastigotes, but not promastigotes, show a growth defect

**Fig. 2:**
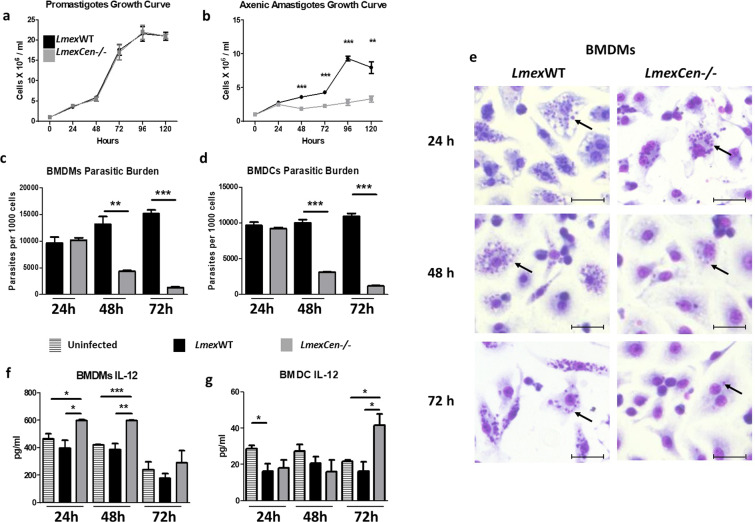
*LmexCen*^*−/−*^ parasites show diminished survival and virulence in bone-marrow-derived macrophages and dendritic cells. **a, b** Growth curve of *LmexWT* and *LmexCen*^*−/−*^ promastigotes (**a**) and axenic amastigotes (**b**). **c**, **d** Parasitic burden in BALB/c-derived BMDMs (**c**) and BMDCs (**d**) infected with either *LmexWT* or *LmexCen*^*−/−*^ promastigotes. **e** Representative Giemsa stain images of BMDMs incubated with either *LmexWT* or *LmexCen*^*−/−*^ promastigotes. Examples of intracellular parasites are indicated by black arrows. Scale bar: 25,000 μm. **f**, **g** IL-12 levels in BALB/c-derived BMDMs (**f**) and BMDCs (**g**) uninfected and infected with either *LmexWT* or *LmexCen*^*−/−*^ promastigotes and then stimulated with 1 μg/ml LPS. Data show one representative experiment out of three independent experiments and show mean ± SEM, *N* = 3 for each group at each time point. Unpaired two-tailed Student’s *t*-test was performed to compare statistical significance at each time point. A *P*-value < 0.05 was considered significant. In all panels * represents *P* ≤ 0.05, ** represents *P* ≤ 0.01, and *** represents *P* ≤ 0.001.

*L. mexicana* WT (*Lmex*WT) and *L. mexicana Cen*^*−/−*^ (*LmexCen*^*−/−*^) parasites in the promastigote or amastigote stage were cultured for 5 days and the number of parasites per ml was counted daily. We found that both *Lmex*WT and *LmexCen*^*−/−*^ promastigotes showed an analogous growth curve (Fig. 2a). However, *LmexCen*^*−/−*^ amastigotes revealed a significant growth defect starting at 48 h (Fig. 2b), compared to *Lmex*WT amastigotes. The number of *LmexCen*^*−/−*^ amastigotes remained practically unchanged throughout the 5 days of culture in the axenic amastigote medium (Fig. 2b).

### *LmexCen*^*−/−*^ parasites show diminished survival and virulence in bone marrow-derived macrophages and dendritic cells and induce IL-12

Bone marrow-derived macrophages (BMDMs) and bone marrow-derived dendritic cells (BMDCs) were infected with *Lmex*WT or *LmexCen*^*−/−*^ parasites and compared to an uninfected control group. At the appropriate time points, cells were stained with Giemsa and the number of intracellular parasites was counted with an inverted microscope. At 24 h post-infection (hpi), the numbers of *Lmex*WT and *LmexCen*^*−/−*^ parasites within BMDMs (Fig. 2c) and BMDCs (Fig. 2d) were similar, but at later time points (48–72 hpi) *LmexCen*^*−/−*^ parasites showed significantly lower survival and replication compared to their WT counterpart. Representative images of BMDM infection with *Lmex*WT or *LmexCen*^*−/−*^ parasites are shown in Fig. 2e. In order to investigate whether lower numbers of intracellular *LmexCen*^*−/−*^ parasites were due to an internalization defect, we infected BMDMs with either *Lmex*WT or *LmexCen*^*−/−*^ parasites and determined the number of intracellular parasites at 30 min, 1 h, 3 h, and 6 h post-infection. We found no significant differences in the number of internalized *LmexCen*^*−/−*^ compared to *Lmex*WT parasites at these early time points, suggesting that *centrin* deficiency does not affect internalization (Supplementary Fig. 1a–b). These results confirm the inability of *LmexCen*^*−/−*^ amastigotes to replicate and establish infection within the host cells.

Furthermore, we evaluated the levels of IL-12, a disease-protective cytokine^34^, after infection and stimulation with LPS. BMDMs exposed to *LmexCen*^*−/−*^ parasites produced significantly higher levels of IL-12 at 24 and 48 hpi, compared to the uninfected and *Lmex*WT-infected groups (Fig. 2f). A similar trend was seen for BMDCs at 72 hpi (Fig. 2g), highlighting a shift to protective innate immunity after *LmexCen*^*−/−*^ exposure.

### *LmexCen*^*−/−*^ parasites do not lead to lesion development in immunocompromised mouse models

**Fig. 3:**
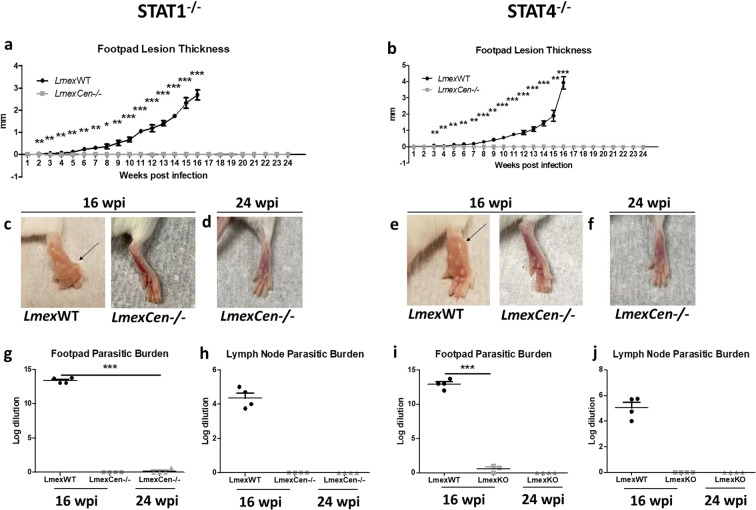
*LmexCen*^*−/−*^ parasites do not lead to lesion development in immunocompromised mice. For this experiment, STAT1^*−/−*^ and STAT4^*−/−*^ female mice were inoculated in the footpad with 10 × 10^6^ *Lmex*WT or *LmexCen*^*−/−*^ promastigotes in the stationary phase. **a**, **b** Lesion thickness in footpad of STAT1^*−/−*^ (**a**) and STAT4^*−/−*^ (**b**) mice. **c**, **e** Representative footpad of STAT1^*−/−*^ (**c**) and STAT4^*−/−*^ (**e**) mice inoculated with *Lmex*WT promastigotes at 16 weeks post infection (wpi). **d**, **f** Representative footpad of STAT1^*−/−*^ (**d**) and STAT4^*−/−*^ (**f**) mice inoculated with *LmexCen*^*−/−*^ promastigotes at 24 wpi. **g**, **i** Footpad parasitic burden of STAT1^*−/−*^ (**g**) and STAT4^*−/−*^ (**i**) mice. **h**, **j** Lymph node parasitic burden of STAT1^*−/−*^ (**h**) and STAT4^*−/−*^ (**j**) mice. Data show one representative experiment out of two independent experiments and show mean ± SEM, *N* = 4 for each group at each time point. Unpaired two-tailed Student’s *t*-test was performed to compare statistical significance at each time point. A *P*-value < 0.05 was considered significant. In all panels * represents *P* ≤ 0.05, ** represents *P* ≤ 0.01, and *** represents *P* ≤ 0.001.

In order to assess the safety of *LmexCen*^*−/−*^ in vivo, we have used signal transducer and activator of transcription (STAT) 1^*−/−*^ and STAT4^*−/−*^ BALB/c immunocompromised models, highly susceptible to *Leishmania* infection. Both STAT1 and STAT4 are key transcription factors involved in Th1 polarization and are critical for the development of a protective immune response against CL^34–37^. STAT1^*−/−*^ and STAT4^*−/−*^ mice were subcutaneously inoculated with either *LmexCen*^*−/−*^ or *Lmex*WT promastigotes in the footpad. STAT1^*−/−*^ and STAT4^*−/−*^ mice infected with *Lmex*WT showed significant footpad swelling starting at 2 weeks post-infection (wpi) for STAT1^*−/−*^ (Fig. 3a), and at 3 wpi for STAT4^*−/−*^ (Fig. 3b). The *Lmex*WT-infected groups reached early removal criteria at 16 wpi due to aggravation of their symptoms. At this time point, we also euthanized a group of mice inoculated with *LmexCen*^*−/−*^ for comparison. We continued monitoring the rest of the *LmexCen*^*−/−*^*-*inoculated mice until 24 wpi to ensure that no cutaneous lesion would present after an initial delay. Remarkably, the groups inoculated with *LmexCen*^*−/−*^ did not show any lesions after 6 months from infection (24 wpi) (Fig. 3a–b). Representative images of footpads infected with *Lmex*WT or *LmexCen*^*−/−*^ at 16 wpi can be found in Fig. 3c for STAT1^*−/−*^ mice and Fig. 3e for STAT4^*−/−*^ mice, while representative images of footpads infected with *LmexCen*^*−/−*^ at 24 wpi can be found in Fig. 3d for STAT1^*−/−*^ mice and Fig. 3f for STAT4^*−/−*^ mice. Furthermore, STAT1^*−/−*^ mice infected with *Lmex*WT showed significantly higher parasitic burdens in their footpad (Fig. 3g) and draining lymph nodes (Fig. 3h), compared to the *LmexCen*^*−/−*^ group, which showed minimal parasitic persistence in the foot and no parasites detectable in the lymph nodes at 16 wpi and 24 wpi. A similar trend was seen in STAT4^*−/−*^ mice (Fig. 3i–j). The lack of symptoms and the limited parasitic persistence in *LmexCen*^*−/−*^ inoculated mice confirm that, as opposed to *Lmex*WT, *LmexCen*^*−/−*^ cannot properly establish and spread the infection, even in a permissive, immunocompromised host.

### *LmexCen*^*−/−*^ parasites do not cause lesions and induce a protective immune response in vivo in BALB/c mice

**Fig. 4:**
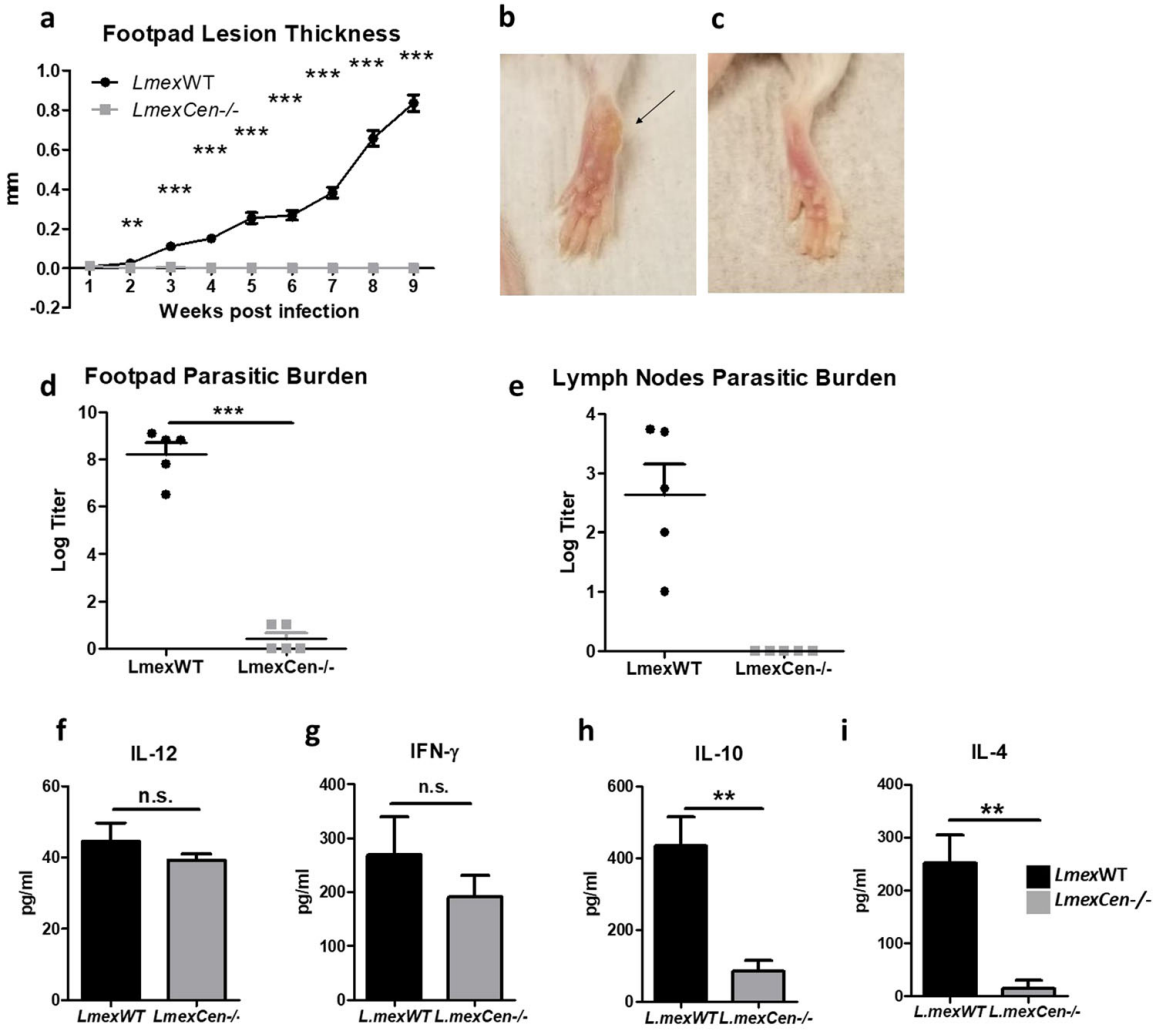
*LmexCen*^*−/−*^ parasites do not cause lesions and induce a protective immune response in vivo. For this experiment, BALB/c female mice were inoculated in the footpad with 2 × 10^6^ *Lmex*WT or *LmexCen*^*−/−*^ promastigotes in the stationary phase. **a** Lesion thickness in the infected footpad. **b**, **c** Representative pictures of footpad inoculated with either *Lmex*WT (**b**) or *LmexCen*^*−/−*^ (**c**) promastigotes at 9 wpi. **d**, **e** Parasitic burden in the footpad (**d**) and draining lymph nodes (**e**) at 9 wpi. **f**–**i** Cytokine levels of IL-12 (**f**), IFN-γ (**g**), IL-10 (**h**), and IL-4 (**i**) in the draining lymph nodes at 9 wpi. Data show one representative experiment out of two independent experiments and show mean ± SEM, *N* = 5 for each group at each time point. Unpaired two-tailed Student’s *t*-test was performed to compare statistical significance at each time point. A *P*-value < 0.05 was considered significant. In all panels * represents *P* ≤ 0.05, ** represents *P* ≤ 0.01, and *** represents *P* ≤ 0.001.

Next, we investigated whether inoculation with *LmexCen*^*−/−*^ parasites resulted in pathological and immunological differences compared to *Lmex*WT infection in susceptible immunocompetent mice. BALB/c mice were inoculated subcutaneously with either *LmexCen*^*−/−*^ or *Lmex*WT promastigotes in the footpad. Mice infected with *Lmex*WT showed a significant increase in footpad lesion thickness throughout the course of infection, while the group inoculated with *LmexCen*^*−/−*^ did not develop cutaneous lesions (Fig. 4a). Representative images of footpads at 9 wpi with *Lmex*WT or *LmexCen*^*−/−*^ can be found in Fig. 4b, c, respectively. Additionally, the *LmexCen*^*−/−*^-inoculated group displayed significantly lower parasitic burdens in the footpad (Fig. 4d) and no parasites were detectable in the draining lymph nodes (Fig. 4e), compared to *Lmex*WT-inoculated mice at 9 wpi.

To determine whether *LmexCen*^*−/−*^ parasites induce a protective immune response, we measured the levels of IL-12, IFN-γ, IL-10, and IL-4 in the draining lymph nodes at 9 wpi, after stimulation with *L. mexicana* antigen. While there was no difference in the levels of Th1 cytokines such as IL-12 (Fig. 4f) and IFN-γ (Fig. 4g) between the two groups, the levels of Th2 cytokines such as IL-10 (Fig. 4h) and IL-4 (Fig. 4i) were significantly lower in *LmexCen*^*−/−*^ immunized mice compared to *Lmex*WT-infected mice. Furthermore, we found higher levels of IgG1 (Supplementary Fig. 2a) antibody titers in *Lmex*WT-infected mice, but similar IgG2a (Supplementary Fig. 2b) antibody titers in both groups. The IgG2a/IgG1 ratio was lower in the *LmexWT* group, compared to the *LmexCen*^*−/−*^ group, but not statistically significant (Supplementary Fig. 2c). Taken together, these results show that *LmexCen*^*−/−*^ parasites promote protective immunity in vivo.

### Immunization with *LmexCen*^*−/−*^ parasites leads to the generation of central memory T cells

**Fig. 5:**
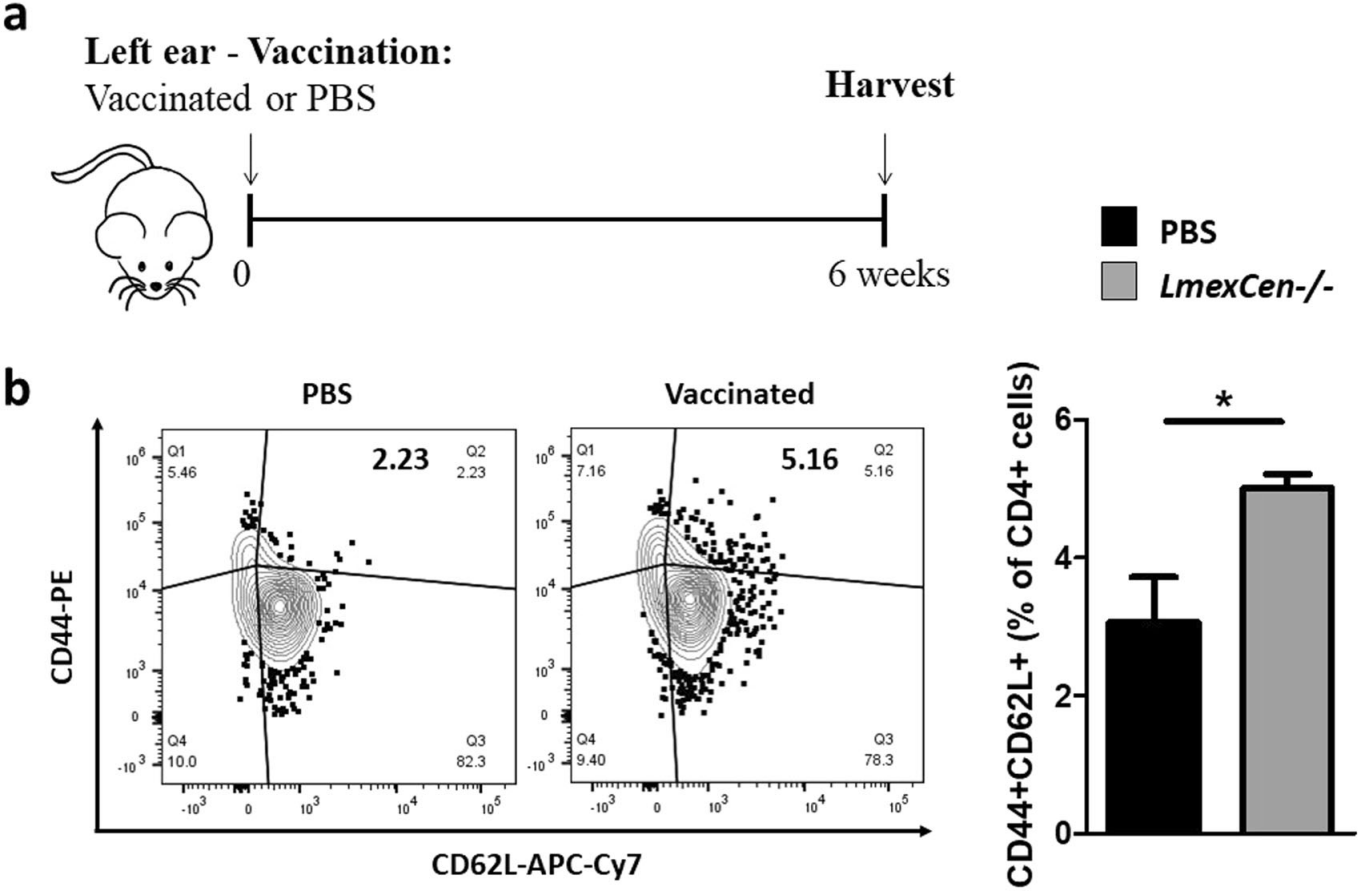
*LmexCen*^*−/−*^ immunization promotes generation of T central memory cells in BALB/c mice. **a** Schematic of the vaccination study design for BALB/c mice. **b** Percentage of CD4^+^CD44^+^CD62L^+^ T cells in the draining lymph nodes of PBS-injected mice and mice immunized with *LmexCen*^*−/−*^ parasites at 6 wpi analyzed via flow cytometry. Data show one representative experiment with mean ± SEM, *N* = 3 for each group. Unpaired two-tailed Student’s *t*-test was performed to compare statistical significance. A *P*-value < 0.05 was considered significant. In all panels * represents *P* ≤ 0.05, ** represents *P* ≤ 0.01, and *** represents *P* ≤ 0.001.

The generation of CD4 + T memory cells following immunization is imperative to maintain protective immunity. In particular, central memory cells homing to the lymph nodes have been shown to mediate long-term immunity to CL^16,17,38^. To assess whether *LmexCen*^*−/−*^ parasites can induce CD4 + central memory T cells (T_CM_), we inoculated either PBS or 1 × 10^6^ stationary phase *LmexCen*^*−/−*^ promastigotes intradermally into the ear of BALB/c mice (Fig. 5a). An intradermal model is physiologically relevant as it simulates the bite of the sand fly vector. We determined the percentage of CD4 + CD44 + CL62L + T cells representing CD4 + T_CM_ cells in the draining lymph nodes at 6 wpi via flow cytometry. We found that mice immunized with *LmexCen*^*−/−*^ parasites displayed a significantly higher percentage of CD4 + CD44 + CL62L + T cells in their lymph nodes, compared to control mice (Fig. 5b). The flow cytometry gating strategy to select CD4 + T cells can be found in Supplementary Fig. 3a. Overall, our results demonstrate that these genetically modified parasites can potentially be used as a vaccination strategy for the protection against *L. mexicana* infection.

### Immunization with *LmexCen*^*−/−*^ parasites mediates protection against *Lmex*WT challenge in BALB/c mice

**Fig. 6:**
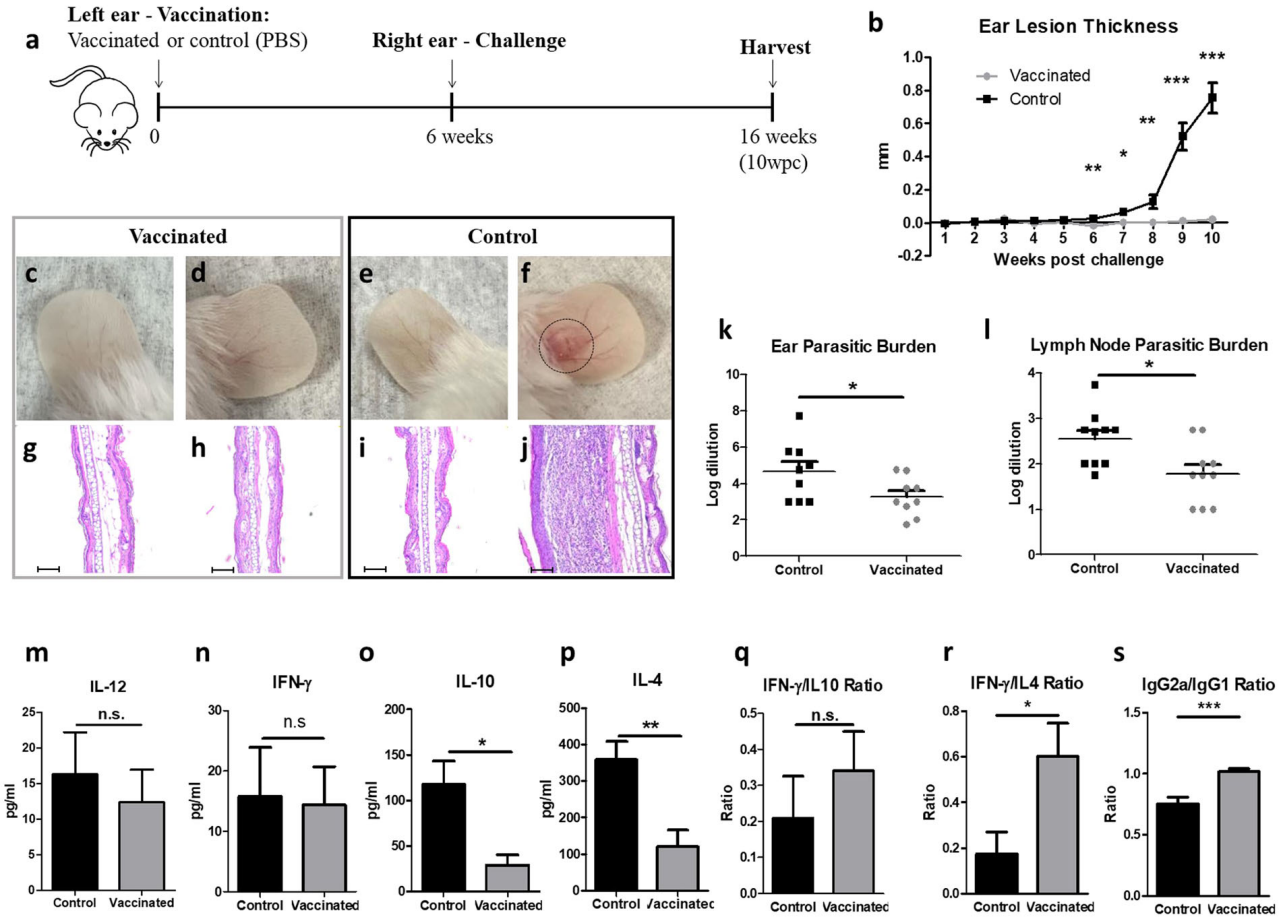
*LmexCen*^*−/−*^ immunization mediates protection against challenge with *Lmex*WT in BALB/c mice. **a** Schematic of the vaccination study design for BALB/c mice. **b** Ear lesion thickness of vaccinated and control mice measured for 10 wpc. **c**–**f** Representative vaccinated ear (**c**), challenged ear of vaccinated mouse (**d**), PBS-inoculated ear of control mouse (**e**), and challenged ear of control mouse (**f**) at 10 wpc. **g**–**j** H&E staining of cross-section of representative vaccinated ear (**g**), challenged ear of vaccinated mouse (**h**), PBS-inoculated ear of control mouse (**i**), and challenged ear of control mouse (**j**) at 10 wpc. Scale bar for **g**–**j**: 100,000 μm. **k**, **l** Parasitic burdens in the ear (**k**) and draining lymph nodes (**l**) at 10 wpc. **m**–**p** Cytokine levels of IL-12 (**m**), IFN-γ (**n**), IL-10 (**o**), and IL-4 (**p**) in the lymph nodes of vaccinated and control mice stimulated with *L. mexicana* antigen at 10 wpc. **q**, **r** Ratio between IFN-γ/IL-10 (**q**) and IFN-γ/IL-4 (**r**) levels in the lymph nodes of vaccinated and control mice at 10 wpc. **s** Ratio of log titers of IgG2a/IgG1 antibodies in the serum of vaccinated and control mice measured for 10 wpc with *Lmex*WT. Data show one representative experiment out of four independent experiments and show mean ± SEM, *N* = 10 for each group at each time point. Unpaired two-tailed Student’s *t*-test was performed to compare statistical significance at each time point. A *P*-value < 0.05 was considered significant. In all panels * represents *P* ≤ 0.05, ** represents *P* ≤ 0.01, and *** represents *P* ≤ 0.001.

To assess the efficacy of *LmexCen*^*−/−*^ parasites, we inoculated either PBS control or stationary phase *LmexCen*^*−/−*^ promastigotes intradermally into the left ear of BALB/c mice. After 6 weeks, both groups were challenged with 1 × 10^4^ *Lmex*WT promastigotes in the stationary phase in the opposite (right) ear. We monitored ear lesions in both groups for an additional 10 weeks post-challenge (wpc) (Fig. 6a).

Starting at 6 wpc, the ear thickness of the control group significantly increased compared to the vaccinated group, which showed no swelling (Fig. 6b). Mice vaccinated with *LmexCen*^*−/−*^ did not develop lesions neither in their vaccinated (Fig. 6c), nor challenged (Fig. 6d) ears at 10 wpc, which resembled the PBS-injected ears of the control group (Fig. 6e). In comparison, the challenged ears of the control group developed large ulcers at 10 wpc (Fig. 6f). Furthermore, the vaccinated (Fig. 6g) and challenged (Fig. 6h) ears of vaccinated mice did not display elevated levels of inflammatory infiltrates, analogous to PBS-injected ears (Fig. 6i). In comparison, the challenged ears of control mice developed substantial inflammation at 10 wpc, as demonstrated by H&E staining (Fig. 6j).

The severity of clinical symptoms in CL is correlated to the parasitic burdens. We found significantly higher parasitic burdens in the ears (Fig. 6k) and draining lymph nodes (Fig. 6l) of control mice compared to the vaccinated group, suggesting that vaccinated mice were able to control the infection. Interestingly, vaccinated mice still showed partial persistence of parasites in both the challenged ears (Fig. 6k) and draining lymph nodes (Fig. 6l) at 10 wpc, which could be important for maintaining a sustained protective immune response.

Immunological characterization revealed that the vaccinated group displayed similar levels of IL-12 (Fig. 6m) and IFN-γ (Fig. 6n), but significantly lower levels of IL-10 (Fig. 6o) and IL-4 (Fig. 6p) in the draining lymph nodes, compared to the control group at 10 wpc. This diminished induction of Th2 cytokines in the vaccinated group resulted in higher ratios of IFN-γ/IL-10 (not significant trend *p* = 0.48) and IFN-γ/IL-4 (statistically significant *p* = 0.03) cytokines in their lymph nodes at 10 wpc compared to the control mice (Fig. 6q–r). Consistent with the cytokine results, we observed a higher ratio of IgG2a/IgG1 antibody titers in the vaccinated group at 10 wpc (Fig. 6s), associated with protective immunity in BALB/c mice^39^. IgG1 and IgG2a antibody titers measurement for the entire duration of the experiment is shown in Supplementary Fig. 2d and e, respectively.

**Fig. 7:**
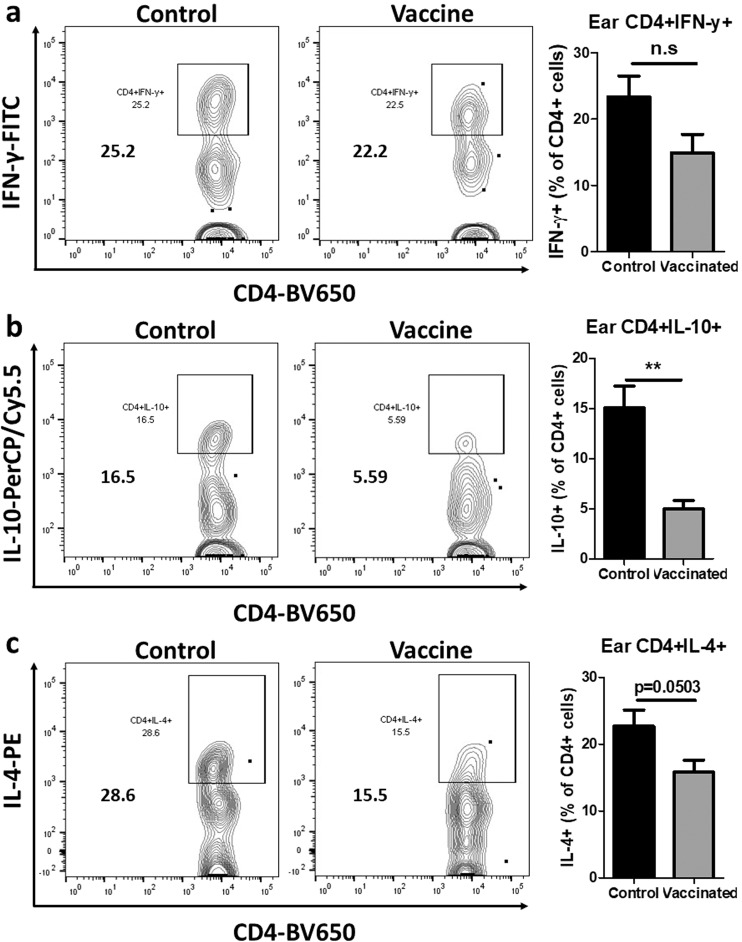
*LmexCen*^*−/−*^ immunization mediates diminished Th2 induction in BALB/c ears challenged with *Lmex*WT. **a** Percentage of CD4^+^IFN-γ^+^ T cells in the ear of vaccinated and control mice at 10 wpc analyzed via flow cytometry. **b** Percentage of CD4^+^IL-10^+^ T cells in the ear of vaccinated and control mice at 10 wpc analyzed via flow cytometry. **c** Percentage of CD4^+^IL-4^+^ T cells in the ear of vaccinated and control mice at 10 wpc analyzed via flow cytometry. Data show one representative experiment out of four independent experiments and show mean ± SEM, *N* = 5 for each group at each time point. Unpaired two-tailed Student’s *t*-test was performed to compare statistical significance. A *P*-value < 0.05 was considered significant. In all panels * represents *P* ≤ 0.05, ** represents *P* ≤ 0.01, and *** represents *P* ≤ 0.001.

To investigate whether a similar immune response characterized by diminished induction of Th2 cytokines could also be found at the infection site, we measured the percentages of CD4^+^IFN-γ^+^, CD4^+^IL-10^+^, and CD4^+^IL-4^+^ T cells in the challenged ears of control and vaccinated mice at 10 wpc (Fig. 7). We found no difference in CD4^+^IFN-γ^+^ percentages (Fig. 7a), but significantly lower percentages of CD4^+^IL-10^+^ T cells (Fig. 7b) and CD4^+^IL-4^+^ T cells (Fig. 7c) in the ears of vaccinated, compared to control mice. The flow cytometry gating strategies and isotype controls are shown in Supplementary Fig. 3b, d and e. Taken all together these results reveal that immunization with *LmexCen*^*−/−*^ parasites is protective against challenges with *Lmex*WT and it induces protective immunity both systemically and locally at the infection site.

### Host genetic background does not affect the efficacy of *LmexCen*^*−/−*^ parasites for the protection against *Lmex*WT

When thinking about advancing a candidate vaccine to human trials, it is important to determine if the genetic background of the host could affect efficacy. To explore this question, we tested whether the protection mediated by *LmexCen*^*−/−*^ parasites in BALB/c mice could be replicated in the genetically distinct C57BL/6 background. While both BALB/c and C57BL/6 mice are susceptible to *L. mexicana*, C57BL/6 mice show a more resistant phenotype, similar to human CL^14,40,41^.

**Fig. 8:**
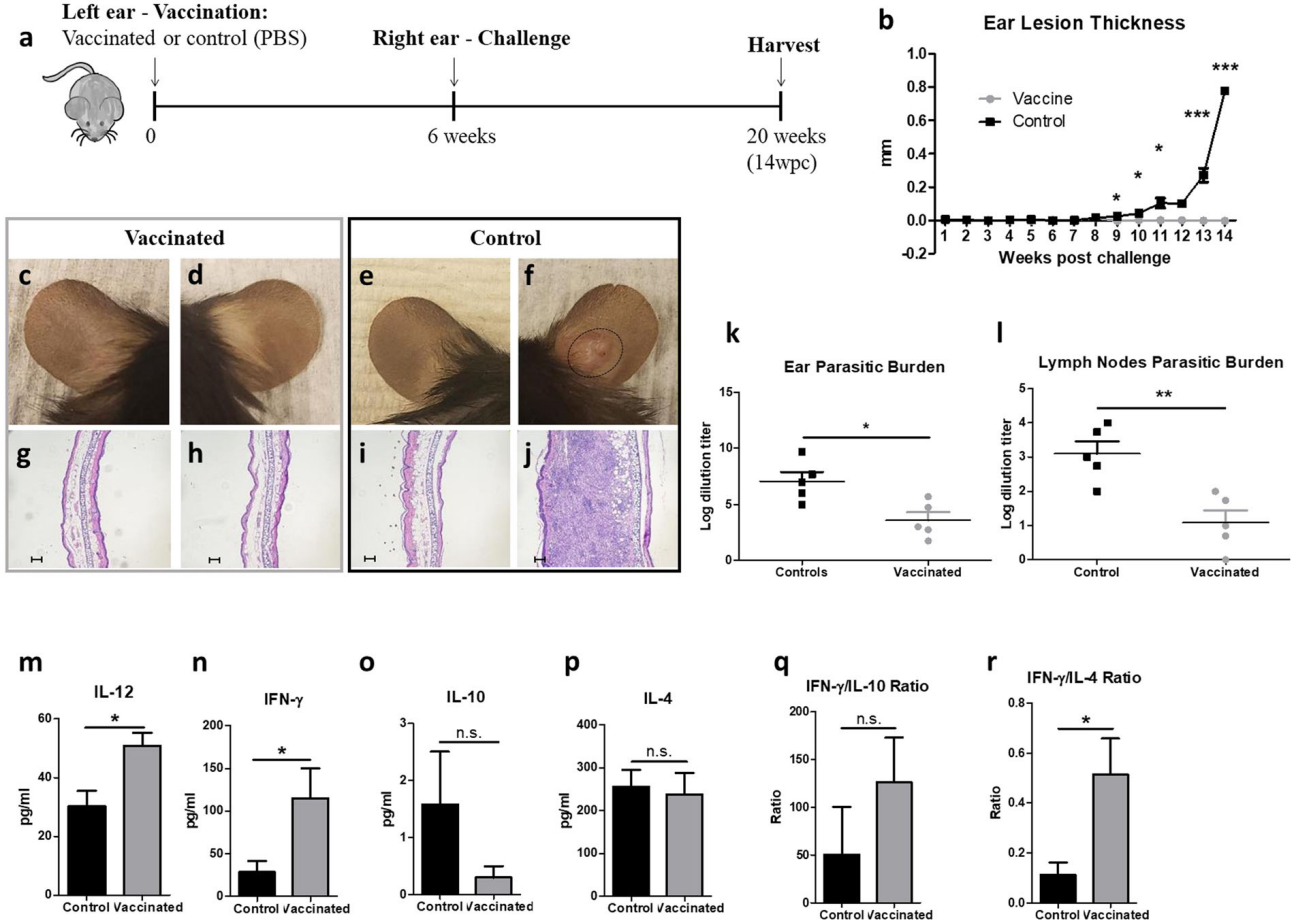
*LmexCen*^*−/−*^ immunization mediates protection against challenge with *Lmex*WT in C57BL/6. **a** Schematic of the vaccination study design for C57BL/6 mice. **b** Ear lesion thickness of vaccinated and control mice measured for 14 wpc. **c**–**f** Representative vaccinated ear (**c**), challenged ear of vaccinated mouse (**d**), PBS-inoculated ear of control mouse (**e**), and challenged ear of control mouse (**f**) at 14 wpc. **g**–**j** H&E staining of cross-section of representative vaccinated ear (**g**), challenged ear of vaccinated mouse (**h**), PBS-inoculated ear of control mouse (**i**), and challenged ear of control mouse (**j**) at 14 wpc. Scale bar for **g**–**j**: 100,000 μm. **k**, **l** Parasitic burdens in the ear (**k**) and draining lymph nodes (**l**) at 14 wpc. **m**–**p** Cytokine levels of IL-12 (**m**) IFN-γ (**n**), IL-10 (**o**), and IL-4 (**p**) in the lymph nodes of vaccinated and control mice stimulated with *L. mexicana* antigen at 14 wpc. **q**, **r** Ratio between IFN-γ/IL-10 (**q**) and IFN-γ/IL-4 (**r**) levels in the lymph nodes of vaccinated and control mice at 14 wpc. Data show one representative experiment out of two independent experiments and show mean ± SEM, *N* = 5 for each group at each time point. Unpaired two-tailed Student’s *t*-test was performed to compare statistical significance at each time point. A *P*-value < 0.05 was considered significant. In all panels * represents *P* ≤ 0.05, ** represents *P* ≤ 0.01, and *** represents *P* ≤ 0.001.

C57BL/6 mice were vaccinated with *LmexCen*^*−/−*^ parasites analogously to BALB/c mice, however, for this experiment we euthanized the mice at 14 weeks post-challenge (wpc), as non-immunized C57BL/6 mice took longer than non-immunized BALB/c mice to develop cutaneous lesions (Fig. 8a). C57BL/6 control mice started developing lesions at 9 wpc, while vaccinated mice did not develop lesions for the whole duration of the experiment (Fig. 8b). Representative images of vaccinated and challenged ears of vaccinated mice at 14 wpc can be found in Fig. 8c and d, respectively, and resemble the PBS-injected ears of the control group (Fig. 8e). In contrast, the challenged ears of control mice displayed cutaneous lesions at 14 wpc (Fig. 8f). Moreover, the vaccinated (Fig. 8g) and challenged (Fig. 8h) ears of vaccinated mice did not reveal elevated levels of inflammatory infiltrates, analogous to the PBS-injected ears (Fig. 8i). On the other hand, the challenged ears of control mice developed substantial inflammation at 14 wpc (Fig. 8j), as shown by H&E staining. Similarly to BALB/c mice, vaccinated C57BL/6 mice showed high IgG1 antibody titers for the entire duration of the experiment (Supplementary Fig. 2f), although IgG2a titers could not be measured as C57BL/6 mice lack the gene that encodes this antibody isotype^42,43^.

At 14wpc the parasitic burden in the ear (Fig. 8k) and draining lymph nodes (Fig. 8l) was significantly lower in the vaccinated, compared to the control mice. This correlated with significantly higher levels of IL-12 (Fig. 8m) and IFN-γ (Fig. 8n) in the draining lymph nodes of vaccinated mice in comparison to the control group. The levels of IL-10 (Fig. 8o) were lower, but not statistically significant in vaccinated mice, while IL-4 levels (Fig. 8p) were comparable between the two groups. This increase in Th1 cytokines in the vaccinated group resulted in higher ratios of IFN-γ/IL-10 (non-significant trend *p* = 0.38) and IFN-γ/IL-4 (statistically significant *p* = 0.02) compared to the non-vaccinated mice (Fig. 8q–r).

**Fig. 9:**
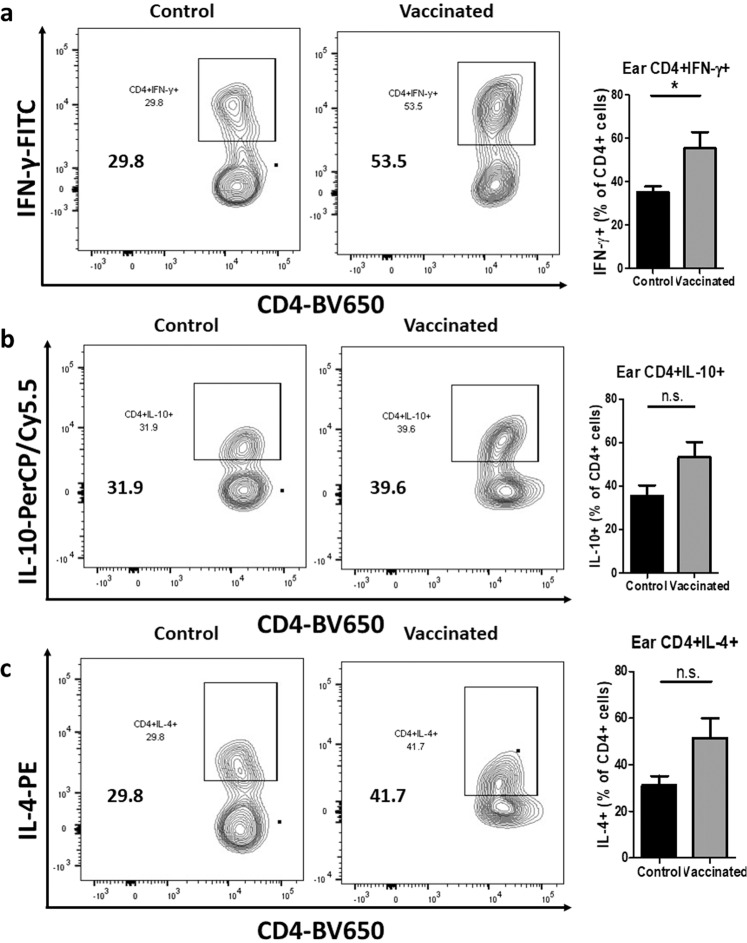
*LmexCen*^*−/−*^ immunization mediates an enhanced Th1 response in C57BL/6 ears challenged with *Lmex*WT. **a** Percentage of CD4^+^IFN-γ^+^ T cells in the ear of vaccinated and control mice at 14 wpc analyzed via flow cytometry. **b** Percentage of CD4^+^IL-10^+^ T cells in the ear of vaccinated and control mice at 14 wpc analyzed via flow cytometry. **c** Percentage of CD4^+^IL-4^+^ T cells in the ear of vaccinated and control mice at 14 wpc analyzed via flow cytometry. Data show one representative experiment out of two independent experiments and show mean ± SEM, *N* = 5 for each group at each time point. Unpaired two-tailed Student’s *t*-test was performed to compare statistical significance. A *P*-value < 0.05 was considered significant. In all panels * represents *P* ≤ 0.05, ** represents *P* ≤ 0.01, and *** represents *P* ≤ 0.001.

An increase in Th1 immunity was also observed in the challenged ears of vaccinated, compared to control mice. In particular, vaccinated mice displayed a significantly higher percentage of CD4 + IFN-γ^+^ (Fig. 9a) T cells in their challenged ears, compared to the control group. Interestingly, no significant difference was observed in the percentage of CD4^+^IL-10^+^ T cells (Fig. 9b) and CD4^+^IL-4^+^ T cells (Fig. 9c) between the groups. The flow cytometry gating strategy and isotype controls are shown in Supplementary Fig. 3c, d, and f. Taken together, these results show that the host genetic background does not affect the overall efficacy of *LmexCen*^*−/−*^ parasites; however, protection is mediated by different immunological mechanisms in BALB/c and C57BL/6 backgrounds. In particular, protection in C57BL/6 mice is mediated by enhanced Th1 responses both systemically and locally at the infection site compared to BALB/c mice where a diminished Th2 response provides protection.

## Discussion

Despite the high morbidity of CL, vector control remains the main preventive strategy against this neglected tropical disease, as there is currently no prophylactic vaccine available for human use^4,8^. Previous studies from our group indicate that *L. major Cen*^*−/−*^ parasites provide protection in cutaneous (against a homologous challenge) and visceral (against heterologous challenge, *L. donovani* infection) leishmaniasis models, showing potential as prophylactic vaccines^25,27^. Nevertheless, *L. major* presents different immunological characteristics and pathologies when compared to the New World *Leishmania* strains causing cutaneous disease^28–30^. Furthermore, *L. major Cen*^*−/−*^ parasites have not yet been tested against American CL, and their efficacy against *L. mexicana* challenge remains unknown. Inducing protection against *L. mexicana* challenge will more likely be achieved with a homologous vaccine, and it is therefore important to develop a vaccine strain specific for American leishmaniasis. Nevertheless, *L. major Cen*^*−/−*^ parasites are currently advancing to clinical trials and could pave the road for more rapid advancement of *LmexCen*^*−/−*^ parasites as a vaccine candidate against American leishmaniasis.

In this study we presented the successful deletion of the *centrin* gene LmxM.22.1410 using the CRISPR/Cas9 technique. While *L. major Cen*^*−/−*^ parasites were previously generated by our group by replacing the *centrin* gene with an oligonucleotide, bypassing the insertion of an antibiotic resistance gene altogether^25^, *LmexCen*^*−/−*^ parasites were generated by first transfecting with an antibiotic resistance gene, which was then removed by a second round of CRISPR. Aside from the absence of antibiotic resistance, our approach is unique as it uses a pLdCN double gRNA CRISPR construct expressing both the Cas9 nuclease and the two gRNAs^44^, as opposed to different constructs for Cas9 and gRNAs, used by other groups for *L. mexicana*^21–23^. Furthermore, other groups have used transient linear DNA, which is degraded in the cell within 2–3 days, whereas in our method the CRISPR plasmid is maintained in the cell to stably express Cas9 and the gRNAs. Once the gene targeting goal is achieved, the pLdCN CRISPR is removed by culturing in G418 free medium.

Our results show that *LmexCen*^*−/−*^ parasites display a growth defect in the amastigote stage leading to attenuated replication within host cells, and do not establish infection both in vitro and in susceptible models in vivo. We have tested the safety characteristics of *LmexCen*^*−/−*^ parasites in STAT1^*−/−*^ and STAT4^*−/−*^ mice, immunocompromised models with impaired Th1 polarization, known to be highly susceptible to *Leishmania* infection^34–37^. We found that *LmexCen*^*−/−*^ parasites did not cause cutaneous lesions long-term in these mice, demonstrating the avirulent nature of the mutant parasites. To our knowledge, the safety properties of *L. mexicana* mutant strains in immunocompromised models have not been tested to the same rigorous standard for other *L. mexicana* genetic mutants. Without causing cutaneous lesions*, LmexCen*^*−/−*^ parasites are able to persist at the site of infection for several weeks. This sustained exposure to the antigen could act as a continuous booster, crucial for maintaining protective immunity against challenge. This is consistent with studies in CL where a persistent infection has been shown to be desirable/required for protective immunity^45^. Failure to provide long-lasting immunity remains a challenge for experimental vaccines against leishmaniasis, and low levels of parasitic persistence might be required to sustain the protection, as sterile immunity can render the host once again susceptible to infection^46^.

In this study, we have tested the efficacy of *LmexCen*^*−/−*^ parasites against *L. mexicana* WT challenge, starting by investigating whether immunization with *LmexCen*^*−/−*^ parasites is able to induce immunological memory. *L. mexicana* infection is known to impair lymph node expansion resulting in reduced T cell numbers in the lymph nodes^33^. Nevertheless, *LmexCen*^*−/−*^ parasites resulted in the generation of CD4 + T central memory cells (CD4 + CD44 + CD62L + ) in the draining lymph nodes, crucial for protection against CL^16,17,38^. Our results are consistent with previous studies in an *L. major-*infection model^17^, where CD4 + T central memory cells populations have been shown to play a role in maintaining protection. CD4 + T central memory cells observed in *LmexCen*^*−/−*^-immunized mice might play a similar role in long term protection during *L. mexicana* infection, and might be implicated in *LmexCen*^*−/−*^-specific immunological memory. Further studies will be necessary to determine the involvement of other T memory cell populations in vaccine-induced protection.

Furthermore, *LmexCen*^*−/−*^-inoculated BALB/c mice did not develop lesions, and presented a comparable Th1 response, but a diminished induction of the Th2 response, compared to *Lmex*WT infection. An analogous immune profile was also observed in BALB/c mice vaccinated with *LmexCen*^*−/−*^ and challenged with *Lmex*WT parasites. Taken together, the data generated with the BALB/c model reveals that the development of lesions was not inhibited by an enhanced Th1 response, but rather by a diminished induction of the Th2 response. This is not surprising as *L. mexicana* infection is known to display a Th2-dependent susceptibility^31^, while in other CL models, such as *L. major* infection, the role of the Th2 response in susceptibility remains controversial^47–49^. In BALB/c mice infected with *L. major*, IL-4 can play an important role in modulating the establishment of a protective Th1 response^47,48^, and the deletion of the IL-4 receptor in DCs leads to a hypersusceptible phenotype^49^. However, in *L. mexicana* infection, it is well established that IL-10 and IL-4 play a crucial role in the susceptibility of BALB/c mice^14,50^. In particular, female BALB/c mice lacking expression of the IL-4 receptor on CD4 + T cells become resistant to *L. mexicana* infection^51^. In a different model (129/Sv × C57BL/6), IL-4 deficiency prevented lesion development during *L. mexicana* infection^52^. These studies highlight that unlike for *L. major* infection, where Th1 responses are determinants of resistance^53^, in *L. mexicana* infection, a dampening of the dominant Th2 response is crucial to confer resistance.

While most mouse models (with the exception of BALB/c) can mount a strong Th1 response and spontaneously resolve *L. major* lesions^53^, the majority of the available wild type murine genetic backgrounds are susceptible to *L. mexicana*^31^. Despite both BALB/c and C57BL/6 models displaying a susceptible phenotype to a physiologically relevant dose of *L. mexicana*, C57BL/6 mice show smaller lesions and lower parasitic burdens compared to BALB/c mice^31^. This correlates with lower levels of Th2 cytokines and higher levels of IFN-γ in C57BL/6, compared to BALB/c mice^31^. Other Th1 cytokines, such as IL-12, are also important for resistance to *L. mexicana* in C57BL/6 mice^32–34^. These enhanced Th1 responses could therefore make the difference between a phenotype of moderate susceptibility in C57BL/6, and high susceptibility in BALB/c mice. We have observed a similar trend when comparing BALB/c and C57BL/6 mice in our *LmexCen*^*−/−*^ efficacy studies. While vaccinated BALB/c mice showed diminished Th2 induction, vaccinated C57BL/6 mice developed enhanced Th1 immunity, compared to their respective control groups. Despite these differences in the underlying immunological mechanism, the ratios between IFN-γ/IL-10 and IFN-γ/IL-4 showed similar trends in both BALB/c and C57BL/6 models. The ratios between IFN-γ/IL-10 and IFN-γ/IL-4 represent the physiological balance between Th1 and Th2 responses, which determines the outcome of the disease and can make a difference between resistance and susceptibility.

We have also evaluated the humoral immune response in BALB/c and C57BL/6 mice. Although antibodies do not typically play a protective role in leishmaniasis^54^, a predominance in IgG2a/IgG1 has been associated with protective immunity in BALB/c mice^39^. We have found a significant increase in IgG2a/IgG1 in vaccinated vs. control BALB/c mice challenged with *Lmex*WT parasites, but no significant difference in IgG2a/IgG1 between BALB/c mice injected with *LmexCen*^*−/−*^ or *Lmex*WT parasites. It is well established that IgG1 and IgG2a are indicative of a Th2 and Th1 response, respectively. However, it is known that Th1 cytokines can also induce IgG3, as well as other isotypes of IgG2, and that Th2 cytokines can also promote IgE^43,55^. Therefore it is possible that IgG1 and IgG2a alone do not fully reflect the induction of Th1 and Th2 responses. In fact, despite showing no significant differences in IgG2a/IgG1 ratios, BALB/c mice injected with *LmexCen*^*−/−*^ parasites displayed significantly lower levels of Th2 cytokines IL-10 and IL-4. Furthermore, IgG2a titers could not be measured in C57BL/6 mice, as this murine background lacks the gene that encodes this antibody isotype^43^, therefore we were unable to provide the IgG2a/IgG1 ratio as for BALB/c mice. Further studies will be necessary to evaluate whether *LmexCen*^*−/−*^ parasites can lead to the production of other antibody classes, subclasses, and isotypes, and to determine how these correlated with immunity against CL.

Taken together, our data show that *LmexCen*^*−/−*^ immunization mediated protection against challenge with *Lmex*WT and promoted a polarization towards protective immunity in these different murine models, demonstrating that the overall efficacy was not affected by the genetic background of the host.

In conclusion, we validate that CRISPR/Cas9-mediated genetic engineering of *Leishmania* parasites is a suitable method for generating live attenuated parasites, which consistently show safety and efficacy in different *Leishmania* infection models and host genetic backgrounds. Due to these advantages and standardization benefits, *Cen*^*−/−*^ *Leishmania* parasites could become a successful leishmaniasis vaccine for human use. With limited preventive strategies for leishmaniasis, the development of a vaccine would dramatically reduce disease incidence and mortality, filling the critical need of many endemic regions across the world.

## Materials and methods

### Mouse strains and parasites

Female BALB/c, C57BL/6, signal transducer and activator of transcription (STAT) 1 knock out (*Stat*1^*−/−*^) and STAT4 knock out (*Stat*4^*−/−*^) BALB/c mice were purchased from Envigo (Harlan laboratories) Indianapolis, IN, USA. All mice were housed at The Ohio State University animal facility, following approved animal protocols and University Laboratory Animal Resources (ULAR) regulations (2010A0048-R3 Protocol). All the experiments were performed using 3–10 age-matched 5–8-week-old female mice per group.

*Leishmania mexicana* (MNYC/B2/62/m379) parasites were maintained by subcutaneous inoculation into the shaved back rumps of 129S6/SvEvTac mice purchased from Taconic Biosciences, Inc. Amastigotes obtained from the draining lymph nodes of infected animals were grown in vitro in complete M199 medium supplemented with 10% fetal bovine serum (FBS), 1% Penicillin/Streptomycin and 1% HEPES at 26 °C to generate stationary phase promastigotes. Axenic amastigotes were generated from promastigotes in vitro by culturing in Schneider’s Drosophila media (pH 5.2) supplemented with 10% FBS and 1% penicillin (20 units/ml)/streptomycin (20 µl/ml) (Axenic amastigote medium) at 33 °C/5% (vol/vol) CO_2_^56,57^.

### Generation of *L. mexicana centrin*^*−/−*^ parasites

The primers and oligonucleotide donor used to generate the *L. mexicana Cen*^*−/−*^ parasites are as follows. Primers used to generate the pLdCN a&b double gRNA CRISPR construct targeting the *centrin* gene locus: Lmex221410a, 5′ATCGAAGACCTTTGTG CCGGTGCATAATGGACTCGTTTTAGAGCTAGAAATAGCAAG; Lmex221410b, 5′ ATCGAAGACCCAAACAACGACGTGAACCTACGCAACACCATGACGAGCTTACTC. Primers used to generate the 584 bp Bleomycin resistance gene donor: Lmex221410BleF, 5′ CGCTTCGTTGCCGGTGCATAATGGAATCTTCATCGGATCGGGTAC; Lmex221410BleR, 5′ AGA AAAAATCTGCCCAACACCGAACTCAGTCCTGCTCCTCGGCCA. Primers used to detect the wild type and deleted *centrin* gene sequence: Lmex221410F1, 5′ CATTCTGCCTA CAGCGTGAA; Lmex221410R1, 5′ CAACATCACCACCCACTGAG; Lmex221410F2, 5′ CAGGGCTGTACGTGATGAGA; Lmex221410R2, 5′ CCCTATCCGAGTTCCACTGA. Primers and oligonucleotide donor used to generate the pLdCN c&d double gRNA CRISPR construct targeting the Bleomycin resistance gene: Blea, 5′ ATCGAAGACCT TTGTCGCGCGGTGAGCACCGGAAGTTTTAGAGCTAGAAATAGCAAG; Bleb, 5′ ATCG AAGACCCAAACGGGTGTTGTCCGGCACCACCATGACGAGCTTACTC; Bledonor 5′ ATGGCCAAGTTGACCAGTGCCGTTCCCTGGCCTGGGTGTGGGTGCGCGG.

### Genome sequence analysis of *LmexCen*^*−/−*^ parasites

Complete genome sequencing of three clones from *LmexCen*^*−/−*^ was performed using an Illumina NextSeq 500 at the sequencing core facility at the Center for Biologics Evaluation and Research (CBER). *LmexCen*^*−/−*^ sequence reads were aligned against *Leishmania mexicana* (MHOM/GT/2001/U1103) strain reference genome (retrieved from www.tritrypdb.org, release v54)^58^ using the Burrows-Wheeler Aligner Maximal Exact Match algorithm (BWA-MEM)^59^. The alignments were converted to BED files using samtools and processed using the bedtools software package^60,61^. The bedtools coverage command was used with the “-d” option in conjunction with the genomic intervals containing the *centrin* genes (+500 bp UTRs) to count the read depth at each position in the coverage of *centrin* genes with a 200 bp window. The bedtools coverage command was used in conjunction with gene coordinates extracted from the gff genomic annotation file (retrieved from www.tritrypdb.org) to compute the percent coverage of each gene. Genes were compared to the coverage of the same genes in a WT isolate to filter out misalignments and technical artifacts. Remaining genes with less than 100 percent coverage manually inspected for a sharp drop-off in coverage (deletion) versus a gradual decline in close proximity to an inverse increase in coverage in a tandem gene (misalignment).

### BMDMs and BMDCs culture and in vitro infection

Bone marrow-derived macrophages (BMDMs) or dendritic cells (BMDCs) were obtained from the femur and tibias of BALB/c mice. After isolation, the bone marrow was cultured with RPMI medium supplemented with 10% fetal bovine serum (FBS), 1% penicillin/streptomycin, 1% HEPES and further complemented with conditioned media from L-929 cells to produce BMDMs or recombinant granulocyte-macrophage colony-stimulating factor (GM-CSF) to produce BMDCs for 7–10 days. For parasite clearance studies, BMDMs and BMDCs were plated in a 24-well plate at a density of 0.5 × 10^6^ per well and infected overnight with stationary phase *LmexCen*^*−/−*^ or wild type *L. mexicana* (*Lmex*WT) promastigotes at a ratio of 1:10 for BMDMs: parasites^26^, and 1:5 for BMDCs: parasites^62^. These ratios result in ~80% of cells being infected after 24 h. Subsequently, extracellular parasites were removed by washing with PBS and new media was applied. At 24, 48, and 72 h, the supernatant was collected for cytokine ELISA, while the attached cells were stained with Giemsa stain for parasitic burdens. For internalization studies, infected BMDMs were stained with Giemsa stain at 30 min, 1 h, 3 h, and 6 h post-infection.

### in vivo infections

For safety studies, aged-matched STAT1^*−/−*^ and STAT4^*−/−*^ mice with a BALB/c background were inoculated subcutaneously in the footpad with 10 × 10^6^ *Lmex*WT or *LmexCen*^*−/−*^ promastigotes in the stationary phase. For safety and immunological studies, wild type BALB/c mice were inoculated subcutaneously in the footpad with 2 × 10^6^ *Lmex*WT or *LmexCen*^*−/−*^ promastigotes in the stationary phase. Footpad thickness was measured weekly after infection.

For vaccination studies, aged-matched BALB/c or C57BL/6 mice were immunized intradermally in the left ear with 1 × 10^6^ *LmexCen*^*−/−*^ promastigotes in the stationary phase or injected with PBS control. After 6 weeks all groups were challenged with 1 × 10^4^ *Lmex*WT parasites intra-dermally in the opposite (right) ear. Ear lesions were measured once a week after the challenge.

### Lesion size measurements

Footpad and ear thickness was measured with a Thickness Gauge (Standard Type) by Teclock (range 0–20 mm, scale interval 0.01 mm). Lesion sizes were measured weekly. The thickness of the non-infected footpad/ear was subtracted from the thickness of the infected footpad/ear for each mouse, to determine lesion thickness.

### Antibody analysis

BALB/c mice were bled through the tail vain once every two weeks, and serum was extracted via centrifugation for antibody ELISA. Medium binding 96-well plates were coated with 5 μg/ml freeze-thawed *L. mexicana* antigen. The antigen was prepared from *L. mexicana* cultures. At confluency, parasites were washed with 1xPBS three times, and then freeze-thawed in liquid N_2_ and in a 37 °C water bath for three cycles. A BCA assay (Pierce™ BCA Protein Assay Kit, Thermo Fisher Scientific) was used to quantify the protein levels. After coating with 5 μg/ml of freeze-thawed *L. mexicana* antigen, the antibody ELISA plates were incubated overnight at 4 °C. After washing with PBS-Tween (0.05% Tween 20 in 1 × PBS, pH 7.4), plates were blocked with 5% milk in PBS/tween and incubated for 1 h at 37 °C. After washing, the samples were added in duplicates to the first well and a serial dilution was performed. The plates were incubated for 2 h at 37 °C before subsequent wash. The plates were then incubated with IgG1 (BD Biosciences Cat#559626, clone X56) or IgG2a (BD Biosciences Cat#553391, clone R19–15) HRP-linked antibodies at a final concentration of 0.2 μl in 1 ml of PBS-FBS (3:1) for 1 h at 37 °C, washed again, and incubated with TMB solution (Fisher Scientific) until color change. The reaction was then stopped with 5% phosphoric acid. A Molecular Devices SpectraMax M3 microplate reader was used to measure absorbance at 450 nm, and the SoftMax Pro software was used to identify antibody titers.

### Parasitic burden

For in vitro infections of BMDMs and BMDCs, at 30 mins, 1, 3, 6, 24, 48, and 72 h, the cells were washed with PBS, fixed with cold methanol and stained with Giemsa stain (Sigma-Aldrich) for 25 mins. Infection was determined by counting the number of amastigotes per 1000 cells via microscopy. For in vivo safety studies, the footpad and draining lymph nodes were extracted at the time of harvest (9 wpi for BALB/c, and 16 or 24 wpi for STAT1^*−/−*^ and STAT4^*−/−*^ mice), homogenized with a cell strainer in 3 ml of Schneider’s Drosophila medium (Gibco, US) supplemented with 20% heat-inactivated FBS and 1% Penicillin/Streptomycin. For in vivo efficacy studies, the ear and draining lymph nodes were also extracted at the time of harvest (10 wpc for BALB/c, and 14 wpc for C57BL/6 mice). The lymph nodes for the vaccine studies were processed the same way, while each ear was separated into two sheets of the dermis, washed in PBS containing 2% Penicillin/Streptomycin, and processed into small pieces. The tissue was then incubated in HBSS (SIGMA) + 2 mM EDTA + 2% FBS + 1% Penicillin/Streptomycin at 37 °C for 30 min shaking (250 rpm). After centrifugation, the cell pellet was incubated with DMEM + 2 mg/ml Collagenase A + 5% FBS + 1% Penicillin/Streptomycin at 37 °C for 1 hr shaking (250 rpm). The enzymatic reaction was then stopped by adding FBS, and the tissue was mashed into a 70μm strainer and washed with DMEM + 20% FBS + 1% Penicillin/Streptomycin. For parasitic burdens, the footpad, lymph node, or ear cell suspensions were serially diluted (20 time dilution) in duplicates across two 96-well plates, so that each sample was diluted across 24 wells^25^. After 7 days of incubation at 26 °C, plates were examined with an inverted microscope at a magnification of ×40. The values reported in the graphs represent the highest log dilution with viable parasites.

### Cytokine profile

Levels of IL-12, in BMDM and BMDC supernatants (in vitro studies) and levels of IL-12, IFN-γ, IL-10, and IL-4 in lymph node culture supernatants (in vivo studies) were determined by sandwich ELISA. For in vitro studies, cells were stimulated with LPS (1 μg/ml) for 24 h after infection and then washed with PBS. After 24, 48, and 72 h, the supernatant was collected for cytokine analysis. For in vivo vaccination studies, mice were euthanized in a CO_2_ chamber and the draining lymph nodes were extracted and homogenized with a cell strainer. Lymph node suspensions were then plated at a density of 3 × 10^6^/ml and stimulated with 20 μg/ml freeze-thawed *L. mexicana* antigen in RPMI medium supplemented with 10% FBS, 1% Penicillin/Streptomycin, and 1% HEPES for 72 h at 37 °C with 5% CO2. After 72 h, the supernatant was collected and used for cytokine ELISA. 96-well plates were coated with primary capture antibody against IL-12 (Biolegend Cat#511802, clone C18.2), IFN-γ (Biolegend Cat#505702, clone R4–6A2), IL-10 (Biolegend Cat#505002, clone JES5–16E3), and IL-4 (BD Biosciences Cat#554434, clone 11B11) at a final concentration of 2 μg/ml, and incubated overnight at 4 °C. Plates were then blocked with PBS + 10% FBS for 2 h at RT and incubated overnight with 50 μL of culture supernatants or recombinant cytokine (standard curve) in duplicates at 4 °C. After washing with PBS-Tween (0.05% Tween 20 in 1× PBS, pH 7.4), the plates were incubated with biotinylated detection antibodies against IL-12 (Biolegend Cat#505302, clone C17.8), IFN-γ (Biolegend Cat#505804, clone XMG1.2), IL-10 (Biolegend Cat#50496, clone JES5-2A5), and IL-4 (BD Biosciences Cat#554390, clone BVD6-24G2) at a final concentration of 1 μg/ml, for 1 hr at RT, washed again, and incubated with streptavidin-conjugated Alkaline Phosphatase for 30 mins at RT in the dark. After washing again, the plates were incubated with PNPP until development. A Molecular Devices SpectraMax M3 microplate reader was used to measure absorbance at 405 nm, and the SoftMax Pro software was used to quantify cytokine levels against the standard curve. All reagents for ELISA were purchased from Biolegend Inc.

### Flow cytometry

Single-cell suspensions generated after enzymatic digestions of the ears were incubated with 1 × 10^6^ T-cell depleted naïve spleen cells (APCs), with 50 µg/ml freeze-thawed *L. mexicana* antigen at 37˚C for 12–14 h in a 12-well plate^25^. For intracellular cytokine staining, cell suspensions were incubated with Cell Activation Cocktail with Brefeldin A (Biolegend) for 4–6 h at 37 °C. After washing with PBS, normal mouse serum was used to block FC receptors. Cells were then stained with monoclonal antibodies coupled to fluorescent dyes against these antigens: BV650-CD4 (Biolegend Cat#100312, clone: RM4-5), and AF-700-CD8 (Biolegend Cat#100730, clone: 53-6.7) (BioLegend, San Diego, CA) (1ul per 1 × 10^6^ cells) and fixed in 4% paraformaldehyde. For intracellular staining, cells were washed with 1x Intracellular Staining Perm Wash Buffer (BioLegend, San Diego, CA) and stained with monoclonal antibodies coupled to fluorescent dyes against these antigens: FITC-IFN-γ (Biolegend Cat#505806, clone: XMG1.2), PerCP/Cy5.5-IL-10 (Biolegend Cat#505027, clone: JES5-16E3), and PE-IL-4 (Biolegend Cat#504103, clone: 11B11) (1ul per 1 × 10^6^ cells). The following isotype controls were used for intracellular staining: Rat IgG1, κ for FITC (Biolegend Cat#400405, clone: RTK2071), Rat IgG2b, κ for PerCP/Cy5.5 (Biolegend Cat#400631, clone: RTK4530), and Rat IgG1, κ for PE (Biolegend Cat#400407, clone: RTK2071) (1ul per 1 × 10^6^ cells).

For the T central memory cell experiment, lymph node cell suspensions were incubated with normal mouse serum, and then stained with BV650-CD4 (Biolegend Cat#100312, clone: RM4-5), AF-700-CD8 (Biolegend Cat#100730, clone: 53–6.7), PE-CD44 (Biolegend Cat#103024, clone IM7), and APC-Cy7-CD62L (Biolegend Cat#104428, clone MEL-14) (1ul per 1 × 10^6^ cells). Flow cytometry was performed with a FACSCelesta Flow Cytometer (BD Biosciences), and analysis was performed with FlowJo software (Tree Star, Inc., Ashland, OR, USA). Doublets were excluded from the analysis.

### Histology

For the vaccination studies, ears were removed after euthanasia and fixed in 4% paraformaldehyde for >72 h. The tissue was processed by Comparative Pathology and Mouse Phenotyping at The Ohio State University. Briefly, tissue sections (5 μM) were cut and stained with hematoxylin and eosin (H&E). The slides were examined under a light microscope for parasites and inflammatory infiltrates. Images were taken using a Leica DMi1 with the Leica Application Suite (LAS) v4.12 software.

### Statistical analysis

All in vitro data show a representative experiment out of three similar independent experiments. All in vivo data presented shows one representative experiment out of 2–4 similar independent experiments with 4–10 mice per group. N represents different biological replicates. Unpaired two-tailed Student’s *t*-test was performed to compare statistical significance. A *P*-value <0.05 was considered significant. In all figures * represents *P* ≤ 0.05, ** represents *P* ≤ 0.01, and *** represents *P* ≤ 0.001. In all figures, error bars represent SEM (standard error of the mean).

### Reporting summary

Further information on research design is available in the Nature Research Reporting Summary linked to this article.

## Supplementary information

Supplementary Figures and Legends

REPORTING SUMMARY

## Data Availability

All relevant data are available in the main text and supplementary information. The *LmexCen*^*−/−*^ sequencing data are available on BioProject (accession code: PRJNA768831). Any additional information can be provided upon reasonable request to the authors.
