# Supplementary material for: Centrin-deficient Leishmania mexicana confers protection against New World cutaneous leishmaniasis
Source: NPJ Vaccines. 2022 Mar 2;7:32. doi: 10.1038/s41541-022-00449-1 (PMC8891280; doi:10.1038/s41541-022-00449-1)
Supplement: Supplementary file 1 — Supplementary Figures and Legends [file 41541_2022_449_MOESM1_ESM.docx]

**SUPPLEMENTARY FIGURES AND LEGENDS**

**
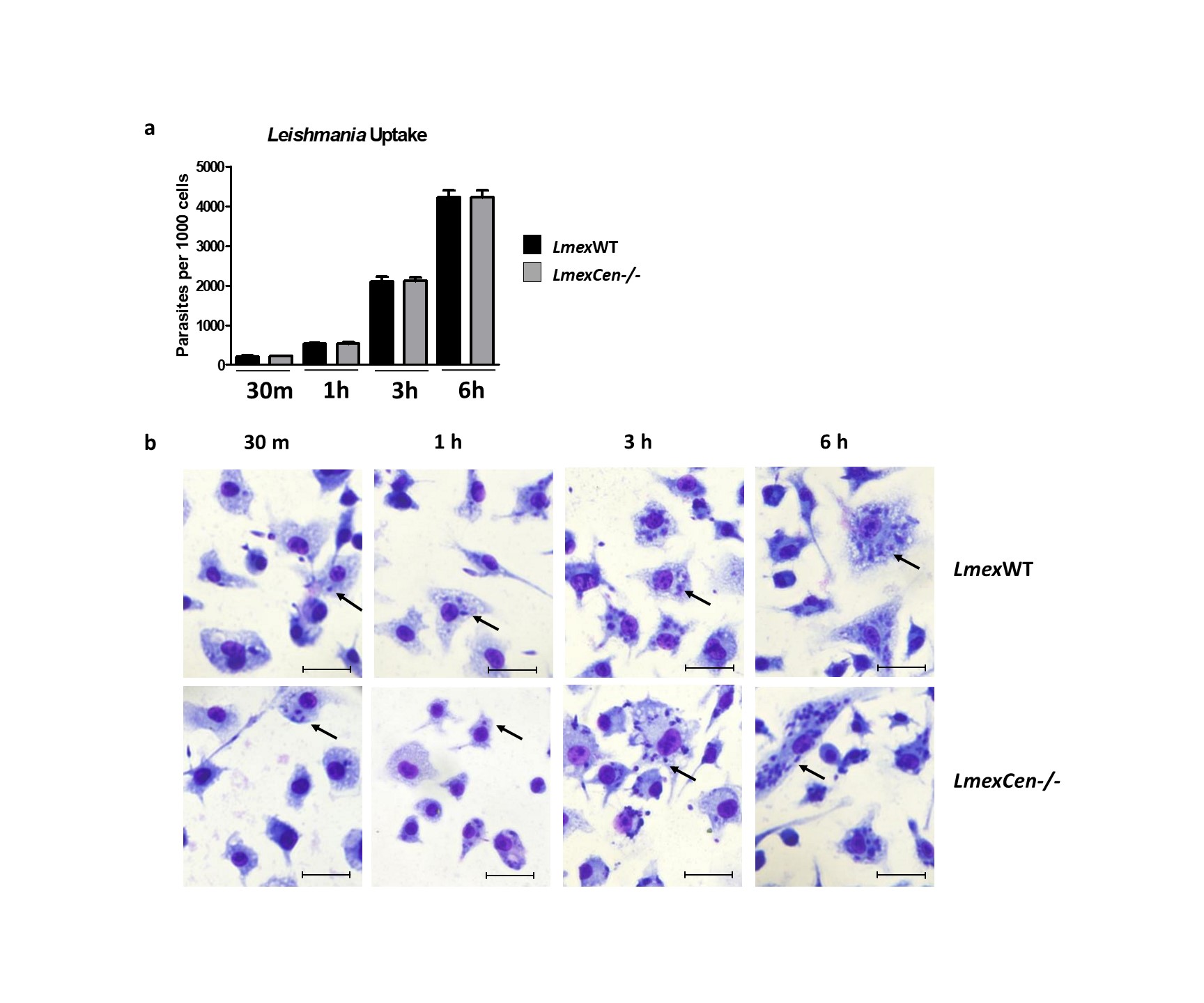
**

**Supplementary Figure 1.** **Internalization of** ***LmexCen^-/-^* and *Lmex*WT parasites in bone marrow-derived macrophages (BMDMs).** **a)** Number of internalized parasites in BALB/c-derived BMDMs infected with either *LmexWT* or *LmexCen^-/-^* promastigotes. **b)** Representative Giemsa stain images of BMDMs incubated with either *LmexWT* (top) or *LmexCen^-/-^* (bottom) promastigotes at early time points. Examples of internalized parasites are indicated by black arrows. Scale bar: 25,000μm. Data show one representative experiment out of two independent experiments and show mean ± SEM, N = 3 for each group at each time point. Unpaired two-tailed Student’s t test was performed to compare statistical significance at each time point. There were no significant differences in this figure.

**
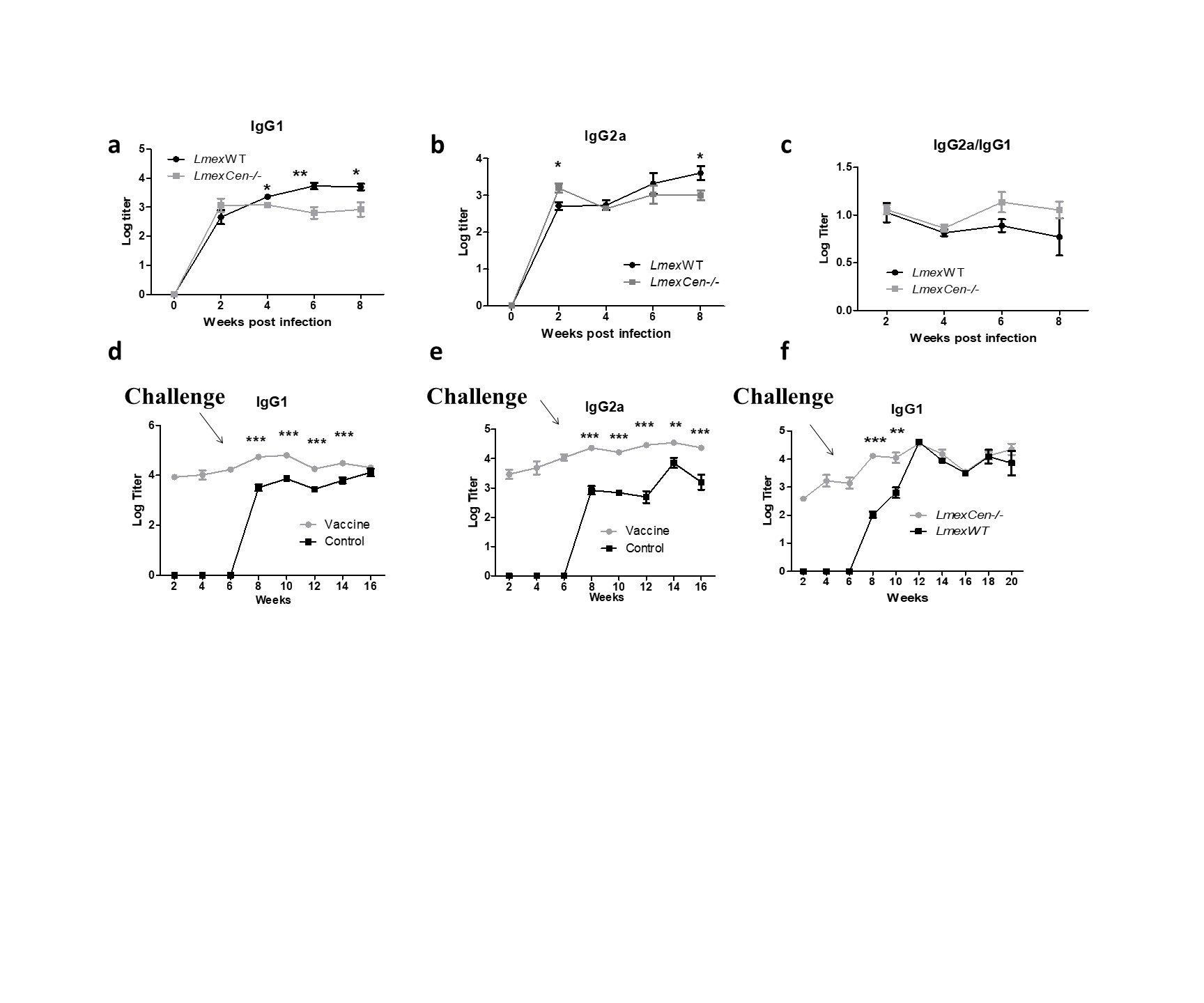
**

**Supplementary Figure 2. Inoculation of *LmexCen^-/-^*** **and *LmexWT* elicits antibody production in BALB/c and C57BL/6 mice.** **a-c**) Log titer of IgG1 (**a**), IgG2a (**b**) and ratio of IgG2a/IgG1 (**c**) antibodies in the serum of BALB/c mice injected with 2 × 10^6^ *LmexCen^-/-^* or *Lmex*WT in the footpad. **d-e**) Log titer of IgG1 (**d**), IgG2a (**e**) antibodies in the serum of vaccinated and control BALB/c mice challenged with *Lmex*WT parasites in the ear. **f**) Log titer of IgG1 antibodies in the serum of vaccinated and control C57BL/6 mice challenged with *Lmex*WT parasites in the ear. Data show one representative experiment out of two independent experiments and show mean ± SEM, N = 5 for each group at each time point for **a-b-c**. Data show one representative experiment out of four independent experiments and show mean ± SEM, N = 10 for each group at each time point for **d-e**. Data show one representative experiment out of two independent experiments and show mean ± SEM, N = 10 for each group at each time point for **f**. Unpaired two-tailed Student’s t test was performed to compare statistical significance at each time point. A P value < 0.05 was considered significant. In all panels * represents P ≤ 0.05, ** represents P ≤ 0.01 and *** represents P ≤ 0.001.

**
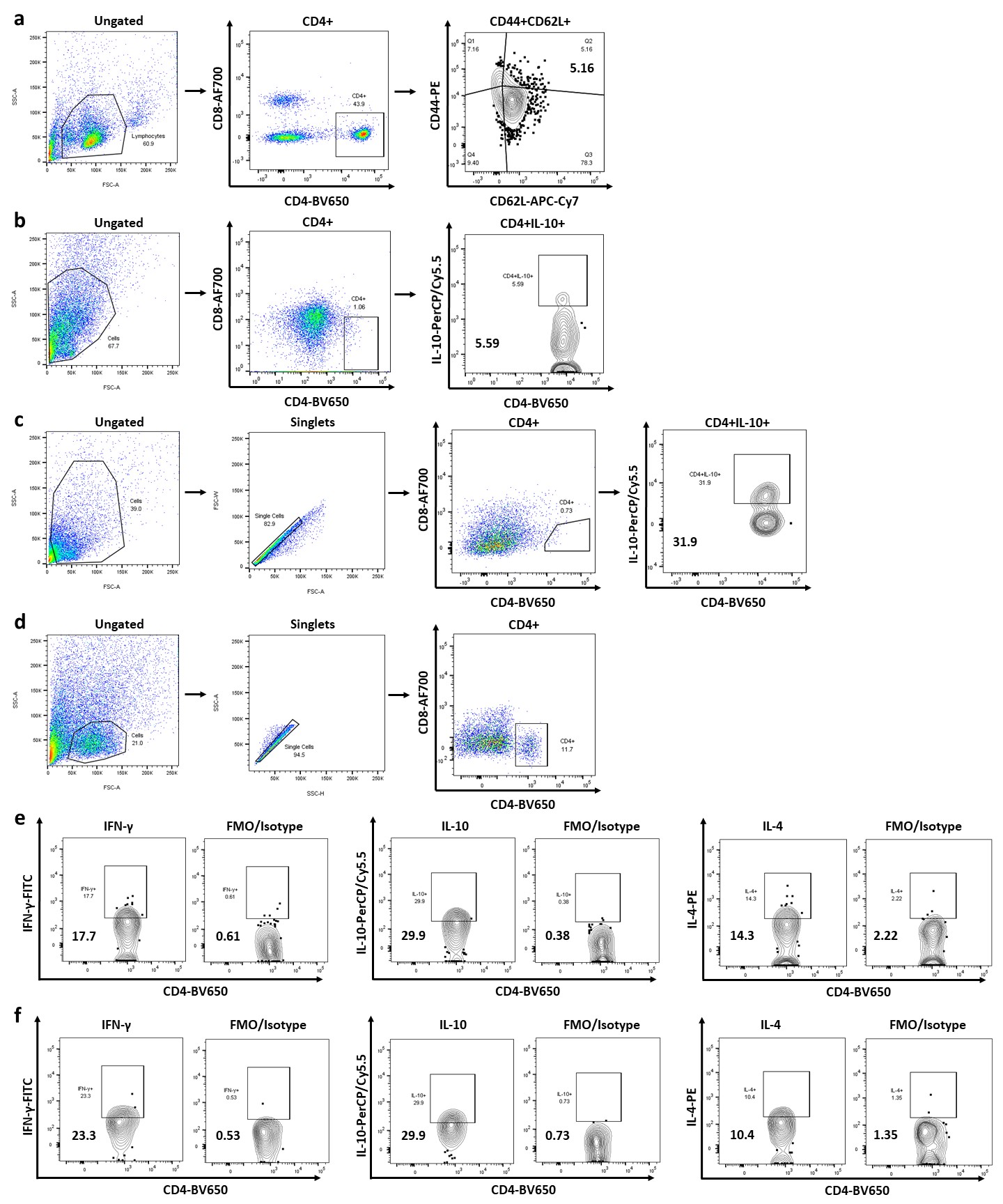
**

**Supplementary Figure 3**. **Flow cytometry gating strategy and controls.** **a**) Representative flow cytometry gating strategy to select CD4+CD44+CD62L+ T cells from lymph node suspensions of BALB/c mice at 6 weeks post vaccination. Data show one representative experiment, N=3 for each group. **b)** Representative flow cytometry gating strategy to determine the cytokine expression by CD4 T cells from ear suspensions of BALB/c mice at 10 wpc. As an example, this panel shows the gating strategy to select CD4+IL-10+ T cells. Data show one representative experiment, N=5 for each group. **c)** Representative flow cytometry gating strategy to to determine the cytokine expression by CD4 T cells from ear suspensions of BALB/c mice at 10 wpc. As an example, this panel shows the gating strategy to select CD4+IL-10+ T cells from ear suspensions of C57BL/6 mice at 14 wpc. Data show one representative experiment, N=5 for each group. **d)** Representative flow cytometry gating strategy to select CD4+ T cells from ear suspensions of BALB/c and C57BL/6 mice for the isotype control experiment (**e-f**). Data show one representative experiment, N=3 for each group. **e)** Flow cytometry positive and FMO controls with isotypes for intracellular staining in the ear of vaccinated and control BALB/c mice at 10 wpc analyzed via flow cytometry. Data show one representative experiment, N=3 for each group. **f)** Flow cytometry positive and FMO controls with isotypes for intracellular staining in the ear of vaccinated and control C57BL/6 mice at 14 wpc analyzed via flow cytometry. Data show one representative experiment, N=3 for each group.
