# Supplementary material for: Centrin-deficient Leishmania mexicana confers protection against New World cutaneous leishmaniasis
Source: NPJ Vaccines. 2022 Mar 2;7:32. doi: 10.1038/s41541-022-00449-1 (PMC8891280; doi:10.1038/s41541-022-00449-1)
Supplement: Supplementary file 2 — REPORTING SUMMARY [file 41541_2022_449_MOESM2_ESM.pdf]

nature portfolio

Corresponding author(s): Abhay Satoskar

Last updated by author(s): Jan 17, 2022

Reporting Summary

Nature Portfolio wishes to improve the reproducibility of the work that we publish. This form provides structure for consistency and transparency in reporting. For further information on Nature Portfolio policies, see our Editorial Policies and the Editorial Policy Checklist.

Statistics

For all statistical analyses, confirm that the following items are present in the figure legend, table legend, main text, or Methods section.

n/a	Confirmed	
☐	☒	The exact sample size (*n*) for each experimental group/condition, given as a discrete number and unit of measurement
☐	☒	A statement on whether measurements were taken from distinct samples or whether the same sample was measured repeatedly
☐	☒	The statistical test(s) used AND whether they are one- or two-sided<br>*Only common tests should be described solely by name; describe more complex techniques in the Methods section.*
☒	☐	A description of all covariates tested
☒	☐	A description of any assumptions or corrections, such as tests of normality and adjustment for multiple comparisons
☐	☒	A full description of the statistical parameters including central tendency (e.g. means) or other basic estimates (e.g. regression coefficient) AND variation (e.g. standard deviation) or associated estimates of uncertainty (e.g. confidence intervals)
☐	☒	For null hypothesis testing, the test statistic (e.g. *F*, *t*, *r*) with confidence intervals, effect sizes, degrees of freedom and *P* value noted<br>*Give P values as exact values whenever suitable.*
☒	☐	For Bayesian analysis, information on the choice of priors and Markov chain Monte Carlo settings
☒	☐	For hierarchical and complex designs, identification of the appropriate level for tests and full reporting of outcomes
☒	☐	Estimates of effect sizes (e.g. Cohen's *d*, Pearson's *r*), indicating how they were calculated

Our web collection on statistics for biologists contains articles on many of the points above.

Software and code

Policy information about availability of computer code

Data collection	Illumina NextSeq 500 using Illumina MiSeq (v2.6.2.1) was used to generate DNA sequence reads.
Data analysis	The SoftMax Pro software was used for antibody and cytokine ELISA. FlowJo 10.7.2. GraphPad Prism version 5.0 was used to generate graphs. Genomic analysis was carried out using bwa v0.7.17, samtools v1.13, bedtools v2.29.2

For manuscripts utilizing custom algorithms or software that are central to the research but not yet described in published literature, software must be made available to editors and reviewers. We strongly encourage code deposition in a community repository (e.g. GitHub). See the Nature Portfolio guidelines for submitting code & software for further information.

Data

Policy information about availability of data

All manuscripts must include a data availability statement. This statement should provide the following information, where applicable:

- Accession codes, unique identifiers, or web links for publicly available datasets
- A description of any restrictions on data availability
- For clinical datasets or third party data, please ensure that the statement adheres to our policy

All relevant data is available in the main text and supplementary information. The LmexCen-/- sequencing data is available on BioProject (accession code: PRJNA768831). Any additional information can be provided upon reasonable request to the authors.

March 2021

Field-specific reporting

Please select the one below that is the best fit for your research. If you are not sure, read the appropriate sections before making your selection.

☒ Life sciences ☐ Behavioural & social sciences ☐ Ecological, evolutionary & environmental sciences

For a reference copy of the document with all sections, see nature.com/documents/nr-reporting-summary-flat.pdf

Life sciences study design

All studies must disclose on these points even when the disclosure is negative.

Sample size	In all experiments we have used at least 3 animals per group to perform student t test analysis; no sample size calculation was necessary or performed since this study is of pre-clinical development stage.
Data exclusions	There was no data exclusions in this manuscript.
Replication	Where possible, all the experiments were replicated 2-4 times using macrophages and dendritic cells from different mice, and different animal models. We confirm that the attempts to replicate the findings are successful.
Randomization	In all experiments animals were randomly allocated between experimental groups.
Blinding	Since this study is of pre-clinical development stage, blinding was not necessary or used in this study.

Reporting for specific materials, systems and methods

We require information from authors about some types of materials, experimental systems and methods used in many studies. Here, indicate whether each material, system or method listed is relevant to your study. If you are not sure if a list item applies to your research, read the appropriate section before selecting a response.

Materials & experimental systems

n/a	Involved in the study	
☐	☒	Antibodies
☐	☒	Eukaryotic cell lines
☒	☐	Palaeontology and archaeology
☐	☒	Animals and other organisms
☒	☐	Human research participants
☒	☐	Clinical data
☒	☐	Dual use research of concern

Methods

n/a	Involved in the study	
☒	☐	ChIP-seq
☐	☒	Flow cytometry
☒	☐	MRI-based neuroimaging

Antibodies

Antibodies used	Flow Cytometry: BV650-CD4 (Biolegend Cat#100312, clone: RM4-5), AF700-CD8 (Biolegend Cat#100730, clone: 53-6.7), FITC-IFN-γ (Biolegend Cat#505806, clone: XMG1.2), PerCP/Cy5.5-L-10 (Biolegend Cat#505027, clone: JES5-16E3), PE-IL-4 (Biolegend Cat#504103, clone: 11B11), FITC Rat IgG1, κ (Biolegend Cat#400405, clone: RTK2071), PerCP/Cy5.5 Rat IgG2b, κ (Biolegend Cat#400631, clone: RTK4530), PE Rat IgG1, κ (Biolegend Cat#400407, clone: RTK2071), PE-CD44 (Biolegend Cat#103024, clone IM7), and APC/Cy7-CD62L (Biolegend Cat#104428, clone MEL-14). Antibody ELISA: IgG1 (BD Biosciences Cat#559626, clone X56), IgG2a (BD Biosciences Cat#553391, clone R19-15). Cytokine ELISA capture antibodies: α-IL-12 (Biolegend Cat#511802, clone C18.2), α-IFN-γ (Biolegend Cat#505702, clone R4-6A2), α-IL-4 (BD Biosciences Cat#554434, clone 11B11), α-IL-10 (Biolegend Cat#505002, clone JES5-16E3). Cytokine ELISA Biotin secondary antibodies: α-IL-12 (Biolegend Cat#505302, clone C17.8), α-IFN-γ (Biolegend Cat#505804, clone XMG1.2), α-IL-4 (BD Biosciences Cat#554390, clone BVD6-24G2), α-IL-10 (Biolegend Cat#50496, clone JES5-2A5).
Validation	The antibodies have been used in previous publications:<br>Flow cytometry: Cell Reports, 2020, PMID: 32023465; Nat Commun. 2019. PMID: 31043609; PNAS, 2020, PMID: 32665441; Cell Metabolism, 2018, PMID: 29874567; Immunity, 2017, PMID: 32187521; Immunity, 2018, PMID: 29625895; Immunobiology, 2020, PMID: 32962814; Cell, 2021, PMID: 34157302; Nature Communications, 2018, PMID: 29867097; Immunity, 2018, PMID: 30527911.<br>Antibody ELISA: Journal of Applied Physiology, 2007, PMID: 17332272; Journal of immunological Methods, 2007, PMID: 17804011<br>Cytokine ELISA: J Immunol. 2021. PMID: 34341171; STAR Protoc. 2021. PMID:34430908; Nat Commun. 2018. PMID: 30093707; Cell Metab. 2020. PMID: 31883840; J Immunol. 2013. PMID: 23514739; Immunity. 2019. PMID: 31231035; Sci Rep. 2019. PMID: 31758034

Eukaryotic cell lines

Policy information about cell lines

Cell line source(s)	Leishmania mexicana (MNYC/BZ/62/M379) used in this study was purchased from ATCC (the American Type culture collection).
Authentication	Certificate of analysis from ATCC.
Mycoplasma contamination	Not tested.
Commonly misidentified lines (See ICLAC register)	No misidentified cell lines were used.

Animals and other organisms

Policy information about studies involving animals; ARRIVE guidelines recommended for reporting animal research

Laboratory animals	Age-matched 5-8 week old female mice were used in our study: C57BL/6 and BALB/c mice; STAT-1KO (BALB/c); STAT-4KO (BALB/c).
Wild animals	No wild animals were used in this study.
Field-collected samples	No field collected samples were used in this study.
Ethics oversight	All mice were housed at The Ohio State University animal facility, following approved animal protocols and University Laboratory Animal Resources (ULAR) regulations (2010A0048-R3 Protocol).

Note that full information on the approval of the study protocol must also be provided in the manuscript.

Flow Cytometry

Plots

Confirm that:

☒ The axis labels state the marker and fluorochrome used (e.g. CD4-FITC).

☒ The axis scales are clearly visible. Include numbers along axes only for bottom left plot of group (a 'group' is an analysis of identical markers).

☒ All plots are contour plots with outliers or pseudocolor plots.

☒ A numerical value for number of cells or percentage (with statistics) is provided.

Methodology

Sample preparation	Please see Materials and methods.
Instrument	Flow cytometry was performed with a FACSCelesta Flow Cytometer (BD Biosciences)
Software	Analysis was performed with FlowJo software (Tree Star, Inc., Ashland, OR, USA)
Cell population abundance	Please see Materials and methods.
Gating strategy	For analysis, Lymphocytes were identified according to their light-scattering properties and doublets were removed using width parameter. CD4+ T-cells were identified as lymphocytes uniquely expressing CD4. Upon further gating intracellular cytokines were measured. Gating strategy for the flow-cytometry analysis is described in Supplementary Figure 3

☒ Tick this box to confirm that a figure exemplifying the gating strategy is provided in the Supplementary Information.
